# Neuropsychological Development of Cool and Hot Executive Functions Between 6 and 12 Years of Age: A Systematic Review

**DOI:** 10.3389/fpsyg.2021.687337

**Published:** 2021-08-11

**Authors:** Laura Fernández García, Ana Merchán, Jessica Phillips-Silver, María Teresa Daza González

**Affiliations:** ^1^Department of Psychology, University of Almería, Almería, Spain; ^2^Center for Neuropsychological Assessment and Rehabilitation (CERNEP), University of Almería, Almería, Spain; ^3^Department of Neuroscience, Georgetown University Medical Center, Washington, DC, United States

**Keywords:** executive functions, neurodevelopment, middle childhood, theory of mind, cognitive control

## Abstract

Previous studies on the development of executive functions (EFs) in middle childhood have traditionally focused on cognitive, or “cool,” EFs: working memory, inhibitory control and cognitive flexibility. However, knowledge of the development of socio-emotional, or “hot,” EFs, such as delay of gratification, decision-making and theory of mind, is more limited. The main aims of this systematic review were to characterize the typical development of both the primary cool and hot EFs in middle childhood, and to identify the main tools for evaluating EFs as a whole. We conducted a systematic search on studies of cognitive and socio-emotional EFs published in the last 5 years in Pubmed, PsycInfo, and WoS databases. Of 44 studies selected, we found a variety of tasks measuring cool EFs, while measures of hot EFs were limited. Nevertheless, the available data suggest that cool and hot components follow distinct, but related, developmental trajectories during middle childhood.

## Introduction

Executive functions (EFs) emerge during childhood and continue to develop into early adulthood (Anderson, 2002). Although multiple definitions of EFs exist in the scientific literature, most authors agree that they refer to the abilities that are necessary for us to carry out goal-directed actions and form adaptive responses to novel or complex situations. In recent years, research on the development of EFs during childhood has increased exponentially, and has shown that the period of middle childhood (ages 6–12 years) is one of significant development, because formal schooling begins, and the demands of the academic and social environments on children are high. Thus, EFs are significant predictors of school readiness, academic achievement, and social behavior (Poon, 2018). For example, school children must be able to maintain an adequate level of attention and motivation, ignore the many distractions that exist in the classroom environment to achieve academic goals, as well as regulate their emotions to adjust their behavior and relate to their peers.

Underlying the development of these behaviors are significant structural and functional brain changes that occur during this time period, which systematically affect cognitive and socioemotional EF abilities at various stages of childhood, as well as their potential for long-term success. For example, in a review of brain development underlying EFs across the lifespan by De Luca and Leventer (2008), the authors point out that during the preadolescent years there is a significant increase in cortical gray matter in frontal areas. This change represents the last major increase in volume in these regions, and seems to reach the highest growth peak at age 11 in girls and 12 in boys (Rapoport et al., 1999). Some neuroimaging studies have found that this increase in cortical gray matter, especially left frontal areas, which correlates with greater working memory ability (Nagy et al., 2004). The available evidence from neuroimaging studies offers insight into widespread changes in neural underpinnings of EFs, which we will address throughout this review.

The literature that has illuminated the importance of EF development during middle childhood comprises many studies, the majority of which have focused exclusively on inhibitory control, working memory and cognitive flexibility—considered by most authors to be the central components of EFs (Diamond, 2013). We describe these components in detail next.

### Working Memory

The majority of studies on EFs address several distinct subcomponents of Baddeley and Hitch's model, which defines WM as a system responsible for simultaneously processing and storing incoming information (1974) (Baddeley and Hitch, 1974). According to this model, WM is composed of (1) verbal WM (or *phonological loop*), which is a passive processing system for verbal and acoustic information, and (2) visuospatial WM (or *visuospatial agenda*), which passively analyzes the information it receives through the visual pathway. Both of these systems are supervised by a third, attentionally-limited control system: (3) the central executive, which oversees manipulation, recall, and processing of information (verbal or visuospatial). Although in the original version of this model visuospatial WM was considered a unitary system, a dissociation between two different subcomponents was later proposed which includes a passive temporary store where static visual information about shape and color is processed (“static visual WM”), and an active test mechanism where dynamic visual information about movement sequences is retained and reviewed (“dynamic spatial WM”; Pickering, 2001).

Recently, a distinction has been made between the central executive functions and an updating function in WM (Engelhardt et al., 2015). Central executive functions refer to the abilities to maintain and manipulate verbal or visuospatial information already stored in WM that exceeds the storage capacity. In contrast, updating refers to the abilities to replace or update current content in WM with new content, as well as to suppress or inhibit content that is no longer relevant according to task demands (Carriedo et al., 2016). Thus, when we talk about WM in the context of cool EFs, we are referring to the processes that involve maintaining, manipulating, and updating primarily auditory and visuospatial information in WM.

### Inhibitory Control

From the age of 3 years, two subtypes of inhibitory control can be differentiated (Gandolfi et al., 2014). The first is the ability to inhibit an automatic or prepotent response. This is required in tasks where a univalent stimulus (e.g., in the *Day-Night Task*, a picture of a sun or a moon) is presented, and a conflict emerges between two response options to the same stimulus (the requirement being either to say the name of the picture, or its opposite). The second is the ability to resist interference from distractors in a conflict task. This ability is required during a task in which stimuli with different features, each associated with a particular response, cause a potential conflict between these features or dimensions, and thus attention must be selectively focused on the relevant cue. The most famous example is the *Stroop Task*, the classic example of which is the conflict that occurs when the meaning of the word (“yellow”) and the color of the letters presented (green) are incongruent, and attention must be focused on the less automatic dimension in order to name the color of the ink, rather than read the word. Interference suppression is thought to be a more complex skill, since both the response conflict and the process of filtering out incongruent information within the stimulus are present (Blasi et al., 2006; Gandolfi et al., 2014).

### Cognitive Flexibility

Cognitive flexibility is thought to consist of two separate processes: “task-switching” and “set-shifting” (Dajani and Uddin, 2015). Task-switching refers to the ability to switch between tasks when different instructions are given for stimuli based on a changing cue. An example is the classic *number–letter task-switching* paradigm, in which participants must respond by categorizing a number/letter stimulus (“2A”) as either a vowel/consonant or odd/even, depending on whether the stimulus is presented in the upper or lower part from the screen (Rogers and Monsell, 1995). In contrast, set-shifting requires shifting attention between different features of the same stimuli according to changing instructions, or shifting between rules within a task. For example, in the *Dimensional Change Card Sorting Task* (DCCS; Zelazo, 2006), participants are presented with a target stimulus that can vary in color (red or blue) and in shape (picture of a rabbit or boat), and two reference stimuli that each share one characteristic with the target (color or shape). Depending on the instruction on each trial (“pay attention to color” or “pay attention to shape”), the participant must focus attention on the indicated characteristic and choose the category where the target stimulus (for example, a red rabbit) matches that characteristic. Thus, participants must shift their attention between the different characteristics of the same stimulus according to the instruction that they are given trial by trial. Some authors point out that set-shifting tasks involve a form of lower-level cognitive flexibility, while task-switching tasks require the most complex form of cognitive flexibility (Bunge and Zelazo, 2006; Dajani and Uddin, 2015).

The trend to focus on these three executive components of EFs has favored a perspective on EFs through a cognitive lens. In recent years, however, research has begun to highlight the importance of emotion and motivation in executive functioning, and to distinguish between cool (cognitive) and hot (socio-emotional) EFs (Zelazo and Carlson, 2012).

According to this distinction, cool EFs are required to solve abstract or decontextualized problems, and are oriented toward the achievement of an objective without any affective, motivational, or social interaction component. In the adult literature, cool EFs are associated with the activation of dorsolateral prefrontal cortex (dlPFC), for example in performing *Go/ No Go Tasks* (Hirose et al., 2012) or *n-back Tasks* (Jonides and Smith, 1997). In contrast, hot EFs are involved in social and affective situations that generate emotion and motivation, as well as tension between immediate gratification and greater long-term reward. In neuroimaging studies of adults, hot EFs have been associated with activity of orbitofrontal and ventromedial regions of prefrontal cortex (OFC and vmPFC, respectively). For example, damage to vmPFC affects adults' performance on the *Iowa Gambling Task* (Bechara et al., 1999), and similar results have been seen in patients performing the *Reversal Learning Task* (Rolls et al., 1994). Using *Delay Discounting Tasks*, some neuroimaging studies have found involvement of OFC, along with dlPFC, in representing the choice value during delay discounting (Massar et al., 2015). These regions have strong connections with the amygdala and other areas of the limbic system associated with emotional processing and regulation of motivation (Happaney et al., 2004).

**Figure 1:**
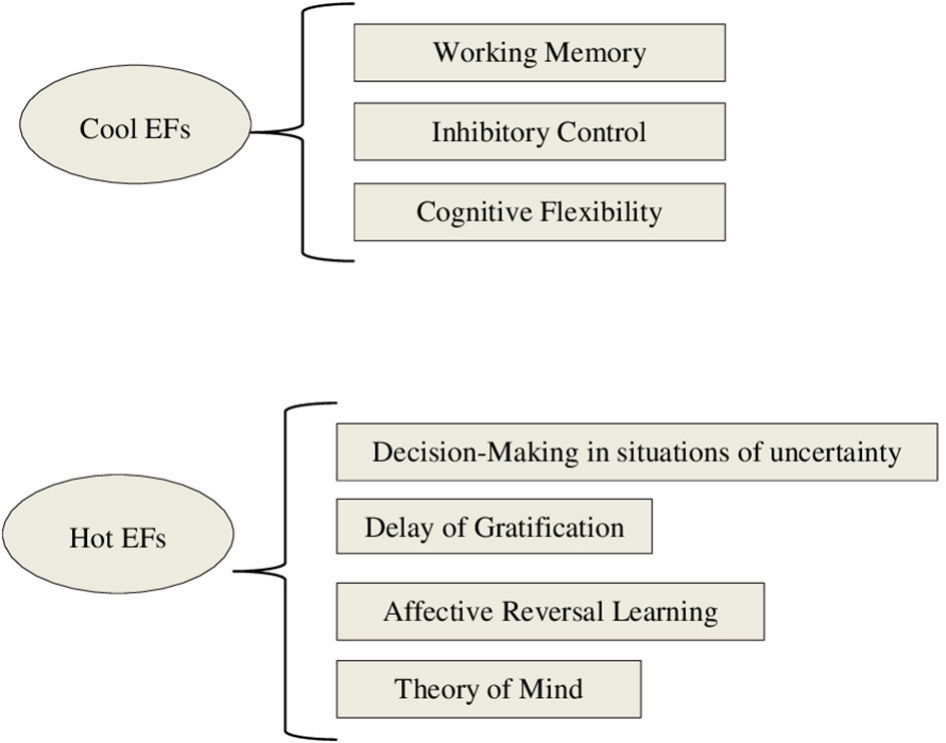
Framework of executive function processes based on Zelazo and Carlson (2012) and Zimmerman et al. (2016).

The current model establishes a clear dissociation between cool and hot EFs (see Figure 1). However, there is growing evidence that cool and hot EFs overlap and, more importantly, form an integrated system (Zelazo and Cunningham, 2007; Tsermentseli and Poland, 2016). For example, researchers have demonstrated that certain regions, such as vlPFC, are recruited both in situations requiring EF that generate emotion, and those that do not involve affective processing (Aron et al., 2004; Zelazo and Carlson, 2012). Furthermore, Zelazo and Cunningham (2007) proposed a model in which emotion regulation, corresponding to the motivational aspect of cognition in conscious, goal-directed problem-solving (as occurrs in hot EFs), is primary or secondary to cognitive components—but cannot be completely isolated from cool EF. Thus, there are conditions under which modulation of emotion is secondary and occurs in the service of solving another problem (e.g., suppressing frustration to be able to muster greater self-control and focus attention on a math lesson). We can say then, that in these types of situations it is necessary to stop and “think cold,” to reflect on them, contextualize them and choose the best response. In this type of situation, it is impossible to distinguish entirely between cool and hot EFs that are involved in regulating our emotions and behavior. According to Zelazo and Cunningham (2007), whether cool or hot EFs are activated more significantly will depend on the motivational significance of the problem, and whether the problem itself is hot or cool.

Despite recent interest in socio-emotional aspects of EFs, there is no clear consensus on what the central components of hot EFs are. The two most widely studied components are decision-making in situations of uncertainty, and delay of gratification or delay discounting—that is, discounting the subjective value of a large reward as the waiting time required to obtain it increases (Zelazo and Carlson, 2012; Peterson and Welsh, 2014). In addition, Zelazo and Carlson (2012) include in the category of hot EFs affective reversal learning—the ability to modify learned associations between stimuli when the contingency relationship between the stimuli changes. This means an individual can learn that a stimulus that was previously followed by a reward now predicts the onset of punishment, and conversely, that a stimulus that was followed by a punishment is now associated with a reward. The implication of the reward system makes this type of learning affective.

Some authors have proposed that other socio-affective abilities should also be included under the umbrella of hot EFs, such as Theory of Mind (ToM) (e.g., Anderson et al., 2008; McDonald, 2013; Tsermentseli and Poland, 2016; Zimmerman et al., 2016). ToM is a heterogeneous and complex construct that refers to the ability to make inferences regarding the mental states (thoughts, beliefs, desires, and emotions) of others. This ability allows individuals to understand that the mental states of others affect their behaviors, thus facilitating the ability to respond appropriately and in accordance with their objectives (Rostan et al., 2014).

The few studies that have analyzed the relationship between ToM and EFs throughout middle childhood suggest that both should follow related developmental trajectories throughout middle childhood and even early adolescence (Marcovitch et al., 2015). Furthermore, although the idea of including ToM as a hot EF is recent, the evidence found in the scientific literature seems to point in this direction. First, neuroimaging studies suggest that during middle childhood one of the main regions involved in tasks in which children must infer that another person does not possess knowledge that they possess is the medial prefrontal cortex (mPFC), a region traditionally associated with the activation of hot EFs (Bowman et al., 2019; Mukerji et al., 2019). Second, some authors who discuss ToM as a hot EF point out the importance of this ability in order to be able to carry out adequate social interactions. For example, ToM allows for sharing cognitive and affective experiences with other people, and predicting the behavior of others in order to regulate one's own behavior and emotions and adapt within the given social context (McDonald, 2013; Zimmerman et al., 2016). Therefore, within an integrative model of cognitive and socio-emotional EFs, ToM is the executive function that allows us to regulate ourselves within a social context, since decision-making, delay of gratification and affective reversal learning does not necessarily involve interaction with others.

A conceptual model of EFs that differentiates between cool and hot components—but also recognizes their integration in behavior—has important implications in both educational and clinical contexts. On the one hand, previous studies have found that cool EFs are significantly related to academic achievement, while hot EFs are more strongly associated with social skills and behaviors (Tsermentseli and Poland, 2016). Yet there is evidence that successful execution of cool EFs can be affected by hot EF abilities. For example, emotion dysregulation due to a child's anxiety can cause deficiencies in various aspects of attention (Pacheco-Unguetti et al., 2010).

On the other hand, the distinction between cool and hot EFs may help to better characterize specific deficits in EFs of children with neurodevelopmental disorders. For example, Zelazo and Müller (2002) suggested that in children with Autism Spectrum Disorder (ASD), the primary deficits are seen in hot EFs such as ToM, with secondary deficits in cool EFs such as cognitive flexibility. In contrast, the opposite pattern is observed in children with Attention Deficit Hyperactivity Disorder (ADHD), who show primary impairments in cool EFs, such as difficulty inhibiting prepotent responses, with the ability to delay gratification reflecting a secondary deficit in hot EFs.

The role of EFs in both cognitive and socio-emotional functioning of children—and their integration—makes it important to better understand how and when cool and hot components of EFs develop, as well as the significant changes in the underlying neural circuitry that occur throughout middle childhood. It is established that cognitive functions develop in parallel with PFC (Best et al., 2009; Otero and Barker, 2014). For example, children and adolescents' WM ability has been linked with the maturation of the lPFC through structural and functional neuroimaging studies (Johson and de Haan, 2011). Regarding socio-emotional functions, one of the most prominent observations is when children and teenagers fail to make advantageous decisions, especially before age 16. Some neuroimaging evidence has been used in support of the idea that this pattern of behavior is related to a disconnect between subcortical reward processing systems and frontal executive control systems in adolescents, such that they are more driven by reward and can show poor decision making in social situations (Otero and Barker, 2014).

The wide range of measures for different components of EFs offers much insight into cognitive and socio-emotional functioning in childhood, but also poses a challenge for synthesizing evidence across EFs and comparing their developmental trajectories (Miyake et al., 2000). Measures of EFs have typically evaluated several distinct, and partially overlapping, cognitive and affective processes at once (Pereira et al., 2018), making it difficult to understand exactly how each component of EF develops, as well as how they ultimately work together as an integrated system. To this end, we found it necessary to provide a systematic review of the research carried out on the development of cognitive and socio-emotional components of executive functioning in middle childhood. The main aims in this systematic review were: (1) to examine which cool and hot executive components are the most studied in neurotypical children between 6 and 12 years of age, (2) to identify the measures that are most sensitive to changes in cool and hot EFs in children during this period of development, and (3) to describe the main developmental changes observed, and determine whether the trajectories of cool vs. hot EFs are already distinguishable during this period of development.

## Methods

### Search Strategy

For this systematic review, an initial general search was performed using PubMed, PsycInfo and Web of Science databases in February 2020. We chose 2015 as the initial year because in the chapter by Peterson and Welsh published in 2014, they made a previous review about how cool and hot EFs developed from pre-school to adolescence. Therefore, we consider that it is from 2015 when we could find and synthesize new information regarding the development of cool and hot EFs. In the key terms that were included in the search, the different cognitive (cool EFs) and socio-emotional (hot EFs) components described above were considered, as well as terms referring to the population of interest. The terms used in the different databases were: (“Child development” OR “middle childhood”) AND (“Executive function” OR “theory of mind” OR “Inhibitory control” OR “working memory” OR “cognitive flexibility” OR “decision making” OR “delay discounting” OR “affective reversal learning”).” Other search limitations were the date range (2015/2020), the age of the sample (“6-12”), studies on humans (“Humans”) and the language of publication (“English; Spanish”). In addition, additional articles were identified by reviewing the references of the original search publications. The process of data extraction followed the recommendations of the protocol proposed by the Cochrane group (Higgins and Green, 2011) and in the protocol of the PRISMA statement (Preferred Reporting Items for Systematic Review and Meta-Analyses; Moher et al., 2009).

### Selection Criteria

The PICOS strategy (Participants, Intervention, Comparison, Outcome, Studies) was used to define the research question with clear inclusion criteria.

This review included studies that apply: (P) to children with typical development without neurological or psychiatric history who are in the age range between 6 and 12 years old, (I) where at least one of the components of EFs were evaluated with performance tasks. In this case, (C) we selected investigations where performance was compared between different age groups, (O) with the aim to assess changes in the components of EFs throughout development, focusing (S) on longitudinal or cross-sectional study designs.

The following papers were excluded: case studies, theses, communications to conferences and studies without peer review. Thus, all studies where only evaluation protocols were described, or those that did not show results regarding the development of executive components, were not considered in this review.

### Data Extraction and Quality Appraisal

The initial search in the databases mentioned above was performed by the first author (LF), and all duplicate articles were removed. Then, studies that met the eligibility criteria were identified based on title and abstract. Next, the full texts of articles were analyzed to confirm that they met the inclusion criteria. When the first author (LF) had doubts about any article, together with the second of this review (AM), they reached an agreement through discussion. The agreement between reviewers for the inclusion and exclusion of the studies was unanimous. Following this process, the first author (LF) extracted the data from the included articles and analyzed it to determine the quality and risk of bias in the studies, and this appraisal process was reviewed by the second author (AM).

The Newcastle-Ottawa Scale (NOS; Wells et al., 2018, adapted from Herzog et al., 2013) was used to assess the quality of the studies. In this version, the quality scores are based on study sample selection, comparability between study groups, and evaluation of results. Maximum scores of 9 or 10 can be given on this scale for cohort studies and cross-sectional studies, respectively. Studies scoring 6 or higher are considered to have high methodological quality (Orton et al., 2014). In this study, a meta-analysis was not carried out because the selected articles, sample types, assessment instruments, and statistical analysis methods used were heterogeneous. The review protocol is registered in the Prospective International Registry of Systematic Reviews (PROSPERO) with the registry number CRD42020181189.

## Results

### Details of Included Studies

**Figure 2:**
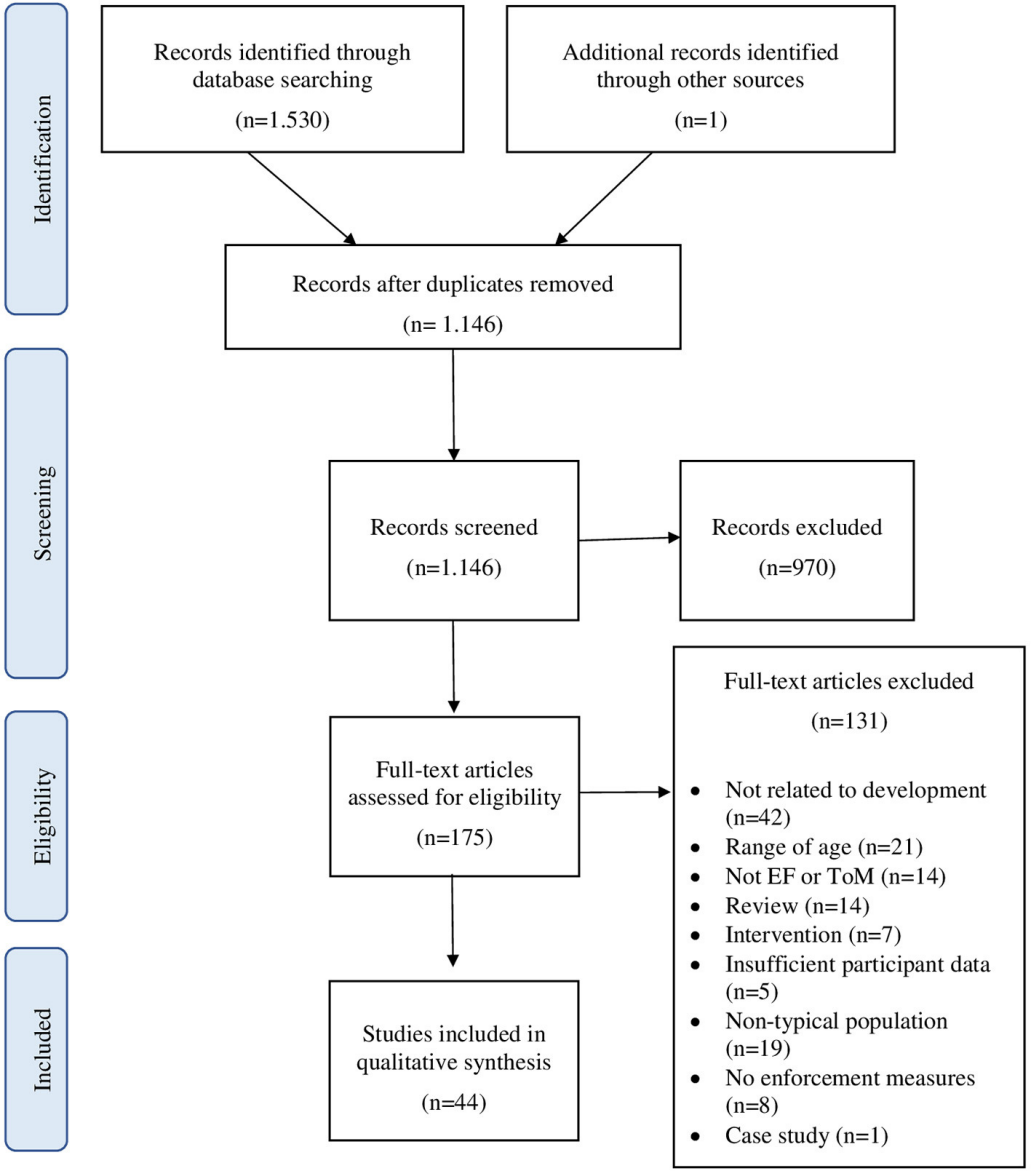
Flow diagram of systematic review.

After searching the databases selected for this study, a total of 1,530 articles were found (621 from PubMed, 456 from Web of Science and 453 PscyInfo). An additional article was also identified through reference list searches and added into the following phases. After removing all duplicate articles (385), a total of 1,146 remained. Based on the title or abstract, 970 articles were excluded, leaving a total of 175 articles. After analyzing the full text of the selected articles to determine if they met the criteria, 131 articles were removed. The reasons to exclude these studies were: the results did not refer to differences in the development of executive components between 6 and 12 years-old (42); the sample was not within the age range of 6 to 12 years-old (21); they did not study any executive component or the theory of mind ability (14); they were bibliographic review articles (14); they focused on studying the effect of a specific intervention on EFs (7); they did not provide sufficient data on the participants (for example, the exact age range of the sample) (5); they included participants with some pathology (19); they did not use performance measures to evaluate EFs (8); and finally, a case study was excluded. Following the study selection process proposed by the PRISMA guidelines, a total of 44 articles were included for this review (see Figure 2).

Of all the studies included in this review, the largest sample consisted of a total of 1,657 participants (Holl et al., 2018), while the study with the smallest sample was 20 participants (Steinbeis et al., 2016).

After analyzing the methodological quality of the studies included in this review based on the method proposed by Orton et al. (2014), we found that 29 articles were evaluated as being of high methodological quality, while the other 15 articles were evaluated as being of low to moderate quality (see Supplementary Table 1 in Supplementary Material).

Regarding our first aim of examining which cool and hot executive components are the most studied in neurotypical children between 6 and 12 years of age, we found that in 34/44 the development of cool EFs was investigated, while in 21/44 the development of hot EFs. In addition, it was also observed that in most studies (33/44), either only the development of cool EFs was analyzed (23/30), or the authors only focused on hot EFs (10/30). Only in 11/44 articles were cool and hot EFs studied in the same age groups.

**Figure 3:**
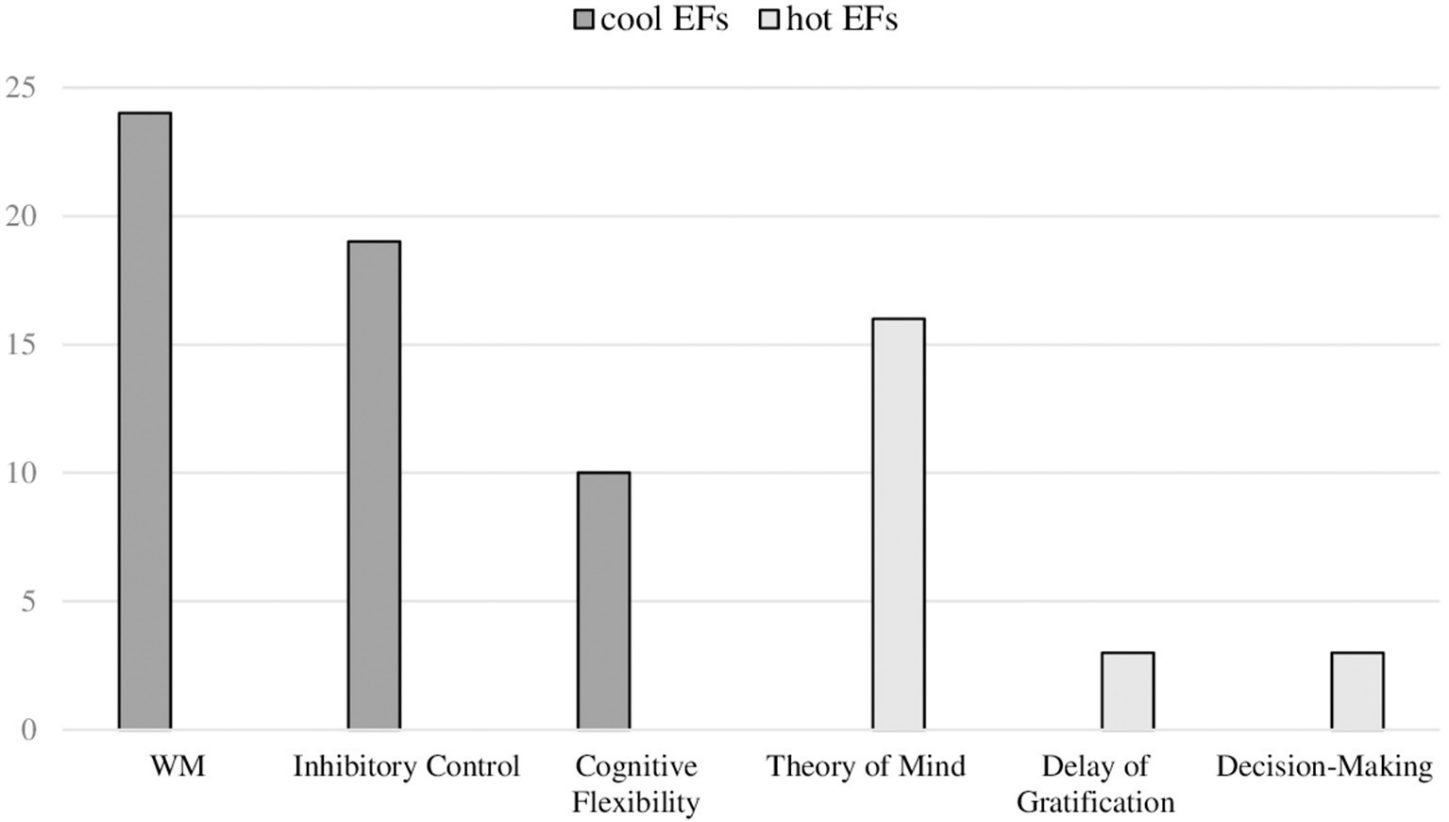
Number of studies included in systematic review, based on cool and hot executive functions analyzed. Working Memory (*n* = 24): (Bock et al., 2015; Kennedy et al., 2015; Kharitonova et al., 2015; Lee Swanson et al., 2015; Rajan and Bell, 2015; Roberts et al., 2015; Bellaj et al., 2016; Lagattuta et al., 2016, 2018; Pailian et al., 2016; Vogan et al., 2016; Barriga-Paulino et al., 2017; Barry et al., 2018; Goriot et al., 2018; Lensing and Elsner, 2018; Ludyga et al., 2018; Matte-Gagné et al., 2018; Nys et al., 2018; Simms et al., 2018; Wilson et al., 2018; Yang and Merrill, 2018; Hoyo et al., 2019; Lecce et al., 2019; Plebanek and Sloutsky, 2019). Inhibitory Control (*n* = 19): (Bock et al., 2015; Kennedy et al., 2015; Rajan and Bell, 2015; Aïte et al., 2016; Bellaj et al., 2016; Lagattuta et al., 2016, 2018; Steinbeis et al., 2016; Symeonidou et al., 2016; Hao, 2017; Mahy et al., 2017; Mous et al., 2017; Arbel et al., 2018; Barry et al., 2018; Goriot et al., 2018; Matte-Gagné et al., 2018; Simms et al., 2018; Wilson et al., 2018; Hoyo et al., 2019). Cognitive Flexibility (*n* = 10): (Bock et al., 2015; Chevalier and Blaye, 2016; Erb et al., 2017; Goriot et al., 2018; Ludyga et al., 2018; Matte-Gagné et al., 2018; Perone et al., 2018; Simms et al., 2018; Wilson et al., 2018; Hoyo et al., 2019). Theory of Mind (*n* = 16): (Bock et al., 2015; Bulgarelli et al., 2015; Chaplin and Norton, 2015; Gómez-Garibello and Talwar, 2015; Kennedy et al., 2015; Lagattuta et al., 2016, 2018; Symeonidou et al., 2016; Wang et al., 2016; Hayward and Homer, 2017; Mahy et al., 2017; Brandone and Klimek, 2018; Holl et al., 2018; Wilson et al., 2018; Hoyo et al., 2019; Lecce et al., 2019). Delay of Gratification (*n* = 3): (Steinbeis et al., 2016; Hao, 2017; Wilson et al., 2018). Decision-Making (*n* = 3): (Audusseau and Juhel, 2015; Almy et al., 2018; Lensing and Elsner, 2018).

We also observed that not all the EFs included in this model that differentiates between cool and hot executive functions were analyzed with the same frequency the reviewed studies. Thus, regarding cool EFs: (1) WM was studied in 24/44 studies; (2) inhibitory control in 19/44; and (3) cognitive flexibility in 10/44. Regarding hot EFs: (1) ToM was investigated in 16/44; (2) delay of gratification in 3/44; and (3) decision-making in 3/44 (see Figure 3). However, no studies were identified that examined the affective reversal learning component of executive functioning, and met the inclusion criteria for this review.

**Figure 4:**
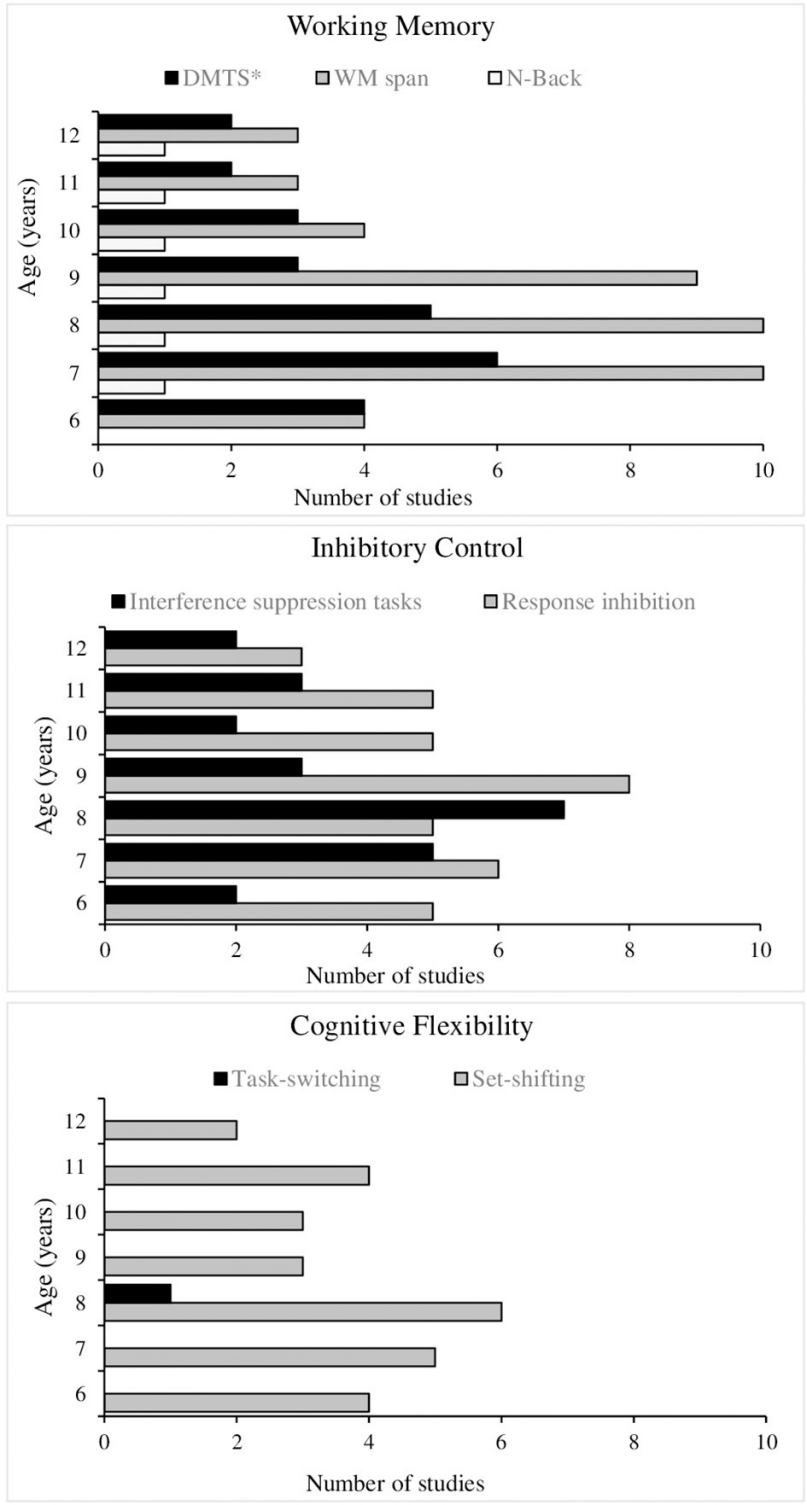
Frequency of task type used to evaluate cool EFs in reviewed studies, by age group.

**Figure 5:**
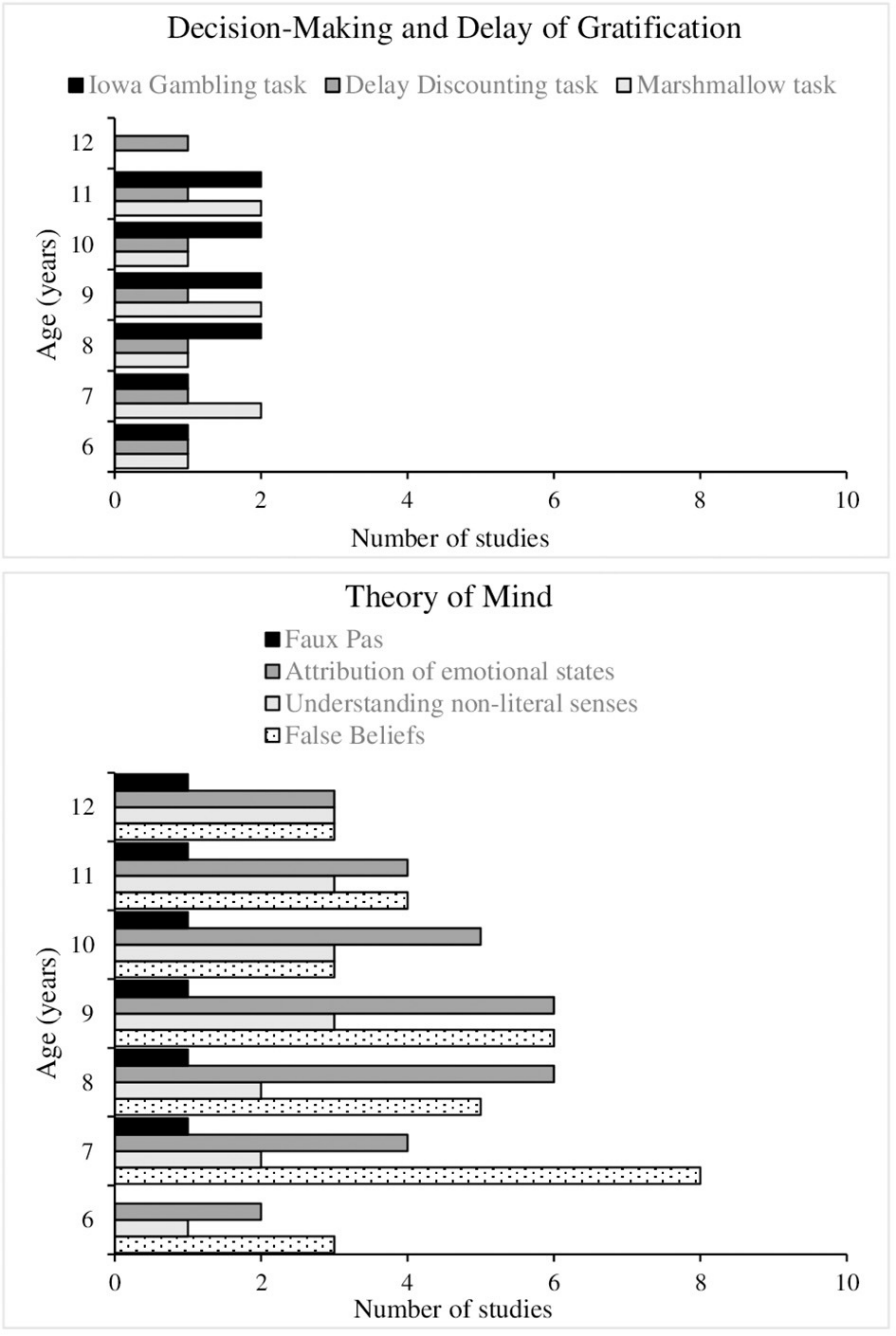
Frequency of task type used to evaluate hot EFs in reviewed studies, by age group.

Following are the results regarding our second and third aims: to identify the measures that are most sensitive to changes in cool and hot EFs in children during middle childhood (see Figures 4, 5 for a summary of the results found), and describe the main developmental changes observed.

### Cool EF Results

#### Working Memory

As mentioned above, WM was the most investigated cool EF in the reviewed studies (24/44). However, not all WM subcomponents were investigated in each of the 24 studies, nor were the same tasks always used.

Verbal WM was investigated in 17/24 studies using 3 different tasks; static visuo-spatial WM in 8/24 studies using 3 different tasks; dynamic visual-spatial WM in 4/24 studies using 2 different tasks; and updating ability only in 2/24 studies with tasks based on the “N-back” paradigm (for a description see Supplementary Table 2 in Supplementary Material).

##### Verbal WM

Only 1/17 studies reviewed used a task based on *DMTS, w*hile the other 16/17 studies used different versions of the *WM span tasks* to assess verbal WM. In 3/16 studies, authors used a task where it was only necessary to actively maintain the information and repeat it in the same order. The remaining 13/16 studies used tasks that required maintaining and manipulating information in WM.

Regarding the developmental results found in this systematic review, of the 17 studies in which verbal WM was evaluated, 13 used a cross-sectional design, and 4 a longitudinal design. All 13 cross-sectional studies found a significant improvement in verbal WM between 6 and 12 years, and 2/4 longitudinal studies found that verbal WM shows significant improvement throughout middle childhood (see Table 1). Of the longitudinal studies, 1/4 observed that the improvement begins to stabilize around 8 years (Matte-Gagné et al., 2018). However, another 2/4 longitudinal studies (Lee Swanson et al., 2015; Roberts et al., 2015), did not find significant improvements in verbal WM ability when they used *WM span tasks* that required maintaining and manipulating information in WM (e.g., *Backward Digit Span task*).

**Table 1:** Studies on the development of the working memory–WM (*n* = 24).

**Subcomponent studied (task)^a^**	**References**	**Design**	**Age groups (*n*)**	**Outcomes**
Verbal WM *(The Letter Matching Task)*	Vogan et al. (2016)	CS	G1 = 12.8 years (*n* = 24) G2 = 22.7 years (*n* = 16)	Significant improvement with age
Verbal WM *(The Memory for Sentences Task)*	Kennedy et al. (2015)	CS	G1 = 4.9 years (*n* = 65) G2 = 7.0 years (*n* = 62) G3 = 9.0 years (*n* = 65)	Significant improvement with age
Verbal WM *(The memory for sentences task)*	Lagattuta et al. (2016)	CS	G1 = 4.10 years (*n* = 63) G2 = 6.11 years (*n* = 66) G3 = 9.4 years (*n* = 87) G4 = 20 years (*n* = 64)	Age was significantly correlated with the performance on WM task
Verbal WM *(The memory for sentences task)*	Lagattuta et al. (2018)	CS	G1 = 4.96 years (*n* = 62) G2 = 7.02 years(*n* = 117) G3 = 9.45 years (*n* = 86) G4 = 20.57years (*n* = 63)	Significant improvement with age
Verbal WM *(Digit Span subtest)* Dynamic visuospatial WM *(The Corsi test)*	Bellaj et al. (2016)	CS	G1 = 7.02 years (*n* = 20) G2 = 7.87 years (*n* = 20) G3 = 8.89 years (*n* = 20) G4 = 9.83 years (*n* = 20) G5 = 10.89 years (*n* = 20) G6 = 12.03 years (*n* = 20)	Significant improvement with age for subcomponents of WM evaluated
Verbal WM *(Digit Span task)* Dynamic visuospatial WM *(Location memory task)*	Bock et al. (2015)	CS	G1 = 7 years (*n* = 42) G2 = 8.5 years (*n* = 35) G3 = 11.5 years (*n* = 27)	Significant improvements after 7 years for subcomponents of WM evaluated
Verbal WM *(Backward Digit Recall subtest from Automated Working Memory Assessment)*	Goriot et al. (2018)	CS	G1 = 4–5 years (*n* = 76) G2 = 8–9 years (*n* = 69) G3 = 11–12 years (*n* = 54)	Age was significantly correlated with the performance on WM task
Verbal WM *(Backward Digit Span Task)*	Hoyo et al. (2019)	CS	G1 = 5.82 years (*n* = 43) G2 = 8.96 years (*n* = 43)	Significant improvement with age
Verbal WM *(Backward Digit Span Task)*	Lecce et al. (2019)	CS	G1 = 9.6 years (*n* = 62) G2 = 10.5 years (*n* = 48) G3 = 11.5 years (*n* = 51) G4 = 12.4 years (*n* = 56)	Significant improvement with age
Verbal WM *(Forward Digit Span and Backward Digit Span)* Static visuospatial WM *(Visual Matrix, and Mapping and Directions)* Updating *(Updating Task)*	Lee Swanson et al. (2015)	LG	T1 = 7.65 years (*n* = 410) T2 = 8.38 years (*n* = 410) T3 = 9.59 years (*n* = 347)	No improvements with age in any subcomponents of WM evaluated
Verbal WM *(Digit span backward task)*	Lensing and Elsner (2018)	LG	T1 = G1:7.35 years (*n* = 621); G2:8.90 years (*n* = 975) T2 = G1:8.35 years (*n* = 596); G2:9.90 years (*n* = 955) T3 = G1:10.35 years (*n* = 565); G2:11.90 years (*n* = 877)	Younger children had slightly more pronounced WM growth curves over time
Verbal WM *(Backward word/digit span)*	Matte-Gagné et al. (2018)	LG	T1 = 1.25 years (*n* = 106) T2 = 2.17 years (*n* = 106) T3 = 6.00 years (*n* = 106) T4 = 7.08 years (*n* = 106) T5 = 7.83 years (*n* = 106) T6 = 8.75 years (*n* = 106)	Significant improvements before stabilizing around 8 years
Verbal WM *(Digit Span Test)* Dynamic visuospatial WM *(Spatial Span Test)*	Nys et al. (2018)	CS	G1 = 7.8 years (*n* = 18) G2 = 9.8 years (*n* = 18) G3 = 22.5 years (*n* = 28)	Significant improvement with age for subcomponents of WM evaluated
Verbal WM *(Forward and Backward Digit Span tasks)*	Rajan and Bell (2015)	CS	G1 = 6 years (*n* = 35) G2 = 8 years (*n* = 37)	Significant improvement with age
Verbal WM (Backwards Digit Recall Subtest)	Roberts et al. (2015)	LG	T1 = 6.6 years (*n* = 281) T2 = 6.7 years (*n* = 389) T3 = 6.9 years (*n* = 472) T4 = 7.2 years (*n* = 643)	No improvements with age
Verbal WM *(Digits Forward, Digits Backward, and Digits Forward Interference)* Static visuospatial WM (dots, dots up, and dots interference) Dynamic visuospatial WM *(Dots Sequence, Dots Sequence Backward, and Dots Sequence Interference)*	Roberts et al. (2018)	CS	G1 = 8.15 years (*n* = 102) G2 = 15.58 years (*n* = 101) G3 = 20.86 years (*n* = 100)	The subcomponent of verbal WM there were significant improvements with age for all tasks The static visual-spatial WM gradually decreased in late adolescence And the dynamic visual-spatial WM showed a decrease in adolescence before increasing again in early adulthood
Verbal WM *(List Sorting Working Memory task)*	Simms et al. (2018)	CS	G1 = 5.5years (*n* = 25) G2 = 7.5 years (*n* = 29) G3 = 8 years (*n* = 10)	Age was significantly correlated with the performance on WM task
Static visuospatial WM *(Visual Delayed Match-to-Sample task)*	Barriga-Paulino et al. (2017)	CS	G1 = 6–26 years (*n* = 165) G1 = 6–9 years (*n* = 31) G2 = 10–13 years (*n* = 32) G3 = 14–17 years (*n* = 32) G4 = 18–21 years (*n* = 32) G5 = 22–26 years (*n* = 38)	Significant improvement with age
Static visuospatial WM *(Working Memory Task)*	Kharitonova et al. (2015)	CS	G1 = 6.92 years (*n* = 20) G2 = 24.7 years (*n* = 20)	Significant improvement with age
Static visuospatial WM *(Flicker Change Detection Task)*	Pailian et al. (2016)	CS	G1 = 3.72 years (*n* = 12) G2 = 4.61 years (*n* = 12) G3 = 5.51 years (*n* = 12) G4 = 6.56 years (*n* = 12) G5 = 7.45 years (*n* = 12) G6 = 8.21 years (*n* = 12) G7 = 19.93 years (*n* = 12)	WM ability increases at 7-years-old reaching adult levels
Static visuospatial WM *(Working Memory Capacity Task)*	Plebanek and Sloutsky (2019)	CS	G1 = 4.54 years (*n* = 28) G2 = 7.47 years (*n* = 29) G3 = - years (*n* = 30)	WM ability increases at 7-years-old reaching adult levels
Static visuospatial WM *(Spatial Working Memory task)*	Wilson et al. (2018)	CS	G1 = 5 years (*n* = 19) G2 = 6 years (*n* = 15) G3 = 7 years (*n* = 22) G4 = 8 years (*n* = 16) G5 = 9 years (*n* = 22) G6 = 10 years (*n* = 16) G7 = 11 years (*n* = 16)	Significant improvement with age
Static visuospatial WM *(Change detection task)*	Yang and Merrill (2018)	CS	G1 = 7.06 years (*n* = 31) G2 = 10.03 years (*n* = 31) G3 = 19 years (*n* = 31)	Significant improvement with age
Updating *(2-Back Task)*	Ludyga et al. (2018)	CS	G1 = 10–12 years (*n* = 89)	Age was significantly correlated with the performance on WM task

*CS, Cross-Sectional; LG, Longitudinal; G, Group; T, Testing Time; WM, Working memory*.

a *For a more detailed description of the tasks used to study the different subcomponents of the WM, see the Supplementary Material*.

##### Static Visuospatial WM

We found that 8/8 studies reviewed used a version of *DMTS* tasks. However, in 7/8 studies, participants only had to maintain information in WM, while 2/8 studies used a task where during the delay participants had to suppress interference from an irrelevant question. There was also 1/8 study in which the authors used a *WM span task* where participants had to maintain and manipulate the information presented.

Among all these studies of static visuospatial WM, 7/8 used a cross-sectional design, and 1/8 was a longitudinal study. The longitudinal study did not find any improvements static visuospatial WM ability between 7 and 9 years, although 5/7 cross-sectional studies did show significant improvements between 6 and 12 years. Moreover, one of these cross-sectional studies that used 3 different types of tasks (Roberts et al., 2018) indicated that this ability continues to improve beyond 12 years and then decreases in late adolescence (after age 20) (see Table 1). There were also 2 cross-sectional studies (Pailian et al., 2016; Plebanek and Sloutsky, 2019), that used simpler *DMTS* tasks (e.g., *Flicker Change Detection task* or *Working Memory Capacity task*), and whose authors indicated that children around 7 years of age show a visual WM ability similar to that of adults.

##### Dynamic Visuospatial WM

To evaluate dynamic visuospatial WM, we found that all of the 4 studies reviewed used *WM span tasks*, where participants were asked to reproduce a sequence of stimuli immediately after presentation (immediate recall), or after delay (delayed recall). In 4/4 studies used different *WM span tasks* that required maintaining and manipulating the information. And also, in 1 of these 4 studies the authors employed two *WM span tasks* both requiring participants only to maintain information in WM. The results found for dynamic visuospatial WM, all studies used a cross-sectional design, and all these studies reviewed pointed to an improvement between 7 and 12 years (see Table 1).

##### Updating Ability of WM

The tasks used in the 2 studies on this ability were based on the “N-back” paradigm (Kirchner, 1958). For these studies about updating of WM, one used a longitudinal design and the other used a cross-sectional design. The results about development of this ability in the cross-sectional study showed that improvements in this ability are significant between 10 and 11 years, while the longitudinal study did not find any improvements between 7 and 9 years (see Table 1).

#### Inhibitory Control

This cool EF was the second most studied in the articles included in this review (19/44), but these 19 did not all analyze the same subcomponents or use the same tasks. In 10/19 the ability to inhibit a pre-potent response was investigated with 2 different types of tasks, and in 9/19 the ability to suppress distractor interference was studied with 3 different types of tasks (for a description see Supplementary Table 2 in Supplementary Material).

##### Inhibition of a Pre-potent Response

In 5/10 studies on this ability, tasks were used in which a particular response that becomes automated, must be inhibited on a few trials when a “stop or no-Go signal” appears. The remaining 5/10 studies used tasks in which participants must inhibit a common prepotent response, like saying the name of a stimulus, and instead to say the opposite (for example, saying “night” when a picture of a sun is presented and; saying “day” when a picture of the moon is showed).

The studies focused on the ability to inhibit a pre-potent response (10/19) used a cross-sectional design, and 8/10 found a significant improvement in this ability between 6 and 12 years of age (see Table 2). In fact, in one study authors found with the *Response Set Task from NEPSY-II-NL battery* that inhibition errors (or commission errors) decreased significantly between 6 and 8 years, stabilizing after 8 years (Mous et al., 2017). This result is congruent with 2/10 studies where authors found that 12- or 14-year-old children showed an ability for response inhibition similar to that of adults (Symeonidou et al., 2016; Wilson et al., 2018), and the results found by Arbel et al. (2018), who did not find significant improvement in a group of children between 8 and 14 years.

**Table 2:** Studies on the development of the Inhibitory Control (*n* = 19).

**Subcomponent studied (task)^a^**	**References**	**Design**	**Age groups (*n*)**	**Outcomes**
Inhibition of a prepotent response *(The Stop-Signal Reaction-Time Task)*	Steinbeis et al. (2016)	CS	G1 = 6.6–12.7 years (*n* = 20)	Better performance on this task was positively correlated with age
Inhibition of a prepotent response *(Stop Signal Task)*	Wilson et al. (2018)	CS	G1 = 5 years (*n* = 19) G2 = 6 years (*n* = 15) G3 = 7 years (*n* = 22) G4 = 8 years (*n* = 16) G5 = 9 years (*n* = 22) G6 = 10 years (*n* = 16) G7 = 11 years (*n* = 16)	Significant improvement with age
Inhibition of a prepotent response *(Computarized Go/No-Go Task)*	Arbel et al. (2018)	CS	G1 = 8.8–9.8 years G2 = 9.9–10.7 years G3 = 10.8–11.9 years G4 = 12–14.2 years (total *n* = 112)	No improvements with age
Inhibition of a prepotent response *(Auditory Go/Nogo Task)*	Barry et al. (2018)	CS	G1 = 8–13 years (*n* = 40)	Better performance on this task was positively correlated with age
Inhibition of a prepotent response *(The Simple Go–No Go Task)*	Symeonidou et al. (2016)	CS	G1 = 11.2 years (*n* = 14) G2 = 16.2 years (*n* = 28) G3 = 23 years (*n* = 23)	Inhibition of prepotent responde ability increases at 12-years-old reaching adult levels
Inhibition of a prepotent response *(Happy–Sad; Day–Night)*	Kennedy et al. (2015)	CS	G1 = 4.9 years (*n* = 65) G2 = 7.0 years (*n* = 62) G3 = 9.0 years (*n* = 65)	No improvements with age
Inhibition of a prepotent response *(Happy–Sad; Day–Night)*	Lagattuta et al. (2016)	CS	G1 = 4.10 years (*n* = 63) G2 = 6.11 years (*n* = 66) G3 = 9.4 years (*n* = 87) G4 = 20 years (*n* = 64)	Age was significantly correlated with the performance on inhibitory control task
Inhibition of a prepotent response *(Happy–Sad; Day–Night)*	Lagattuta et al. (2018)	CS	G1 = 4.96 years (*n* = 62) G2 = 7.02 years(*n* = 117) G3 = 9.45 years (*n* = 86) G4 = 20.57 years (*n* = 63)	Significant improvement with age
Study 1: Inhibition of a prepotent response *(Head–Shoulders–Knees–Toes task; Grass/Snow task)*	Mahy et al. (2017)	CS	Study 1 G1 = 3.57 years (*n* = 20) G2 = 4.39 years (*n* = 31) G3 = 5.58 years (*n* = 18) G4 = 6.53 years (*n* = 19) G5 = 7.39 years (*n* = 18)	Age was significantly correlated with the performance on inhibitory control tasks
Inhibition of a prepotent response *(Response Set Task; Statue Task)*	Mous et al. (2017)	CS	G1 = 6–6.5 years (*n* = 74) G2 = 6.5–7 years (*n* = 104) G3 = 7–7.5 years (*n* = 107) G4 = 7.5–8 years (*n* = 120) G5 = 8–8.5 years (*n* = 202) G6 = 8.5–9 years (*n* = 125) G7 = 9–10 years (*n* = 97)	Significant improvements with age, although after 8 years they seemed to stabilize
Suppression of interference *(Stroop Color–Word Task And; The Simon task)*	Aïte et al. (2016)	CS	G1 = 10.2 years (*n* = 49) G2 = 21.7 years (*n* = 52)	Significant improvement with age
Suppression of interference *(Stroop test; Hayling test)*	Bellaj et al. (2016)	CS	G1 = 7.02 years (*n* = 20) G2 = 7.87 years (*n* = 20) G3 = 8.89 years (*n* = 20) G4 = 9.83 years (*n* = 20) G5 = 10.89 years (*n* = 20) G6 = 12.03 years (*n* = 20)	Significant improvement with age, although the differences were especially significant between 7 and 8 years-old
Suppression of interference *(Color Word Stroop Task)*	Bock et al. (2015)	CS	G1 = 7 years (*n* = 42) G2 = 8.5 years (*n* = 35) G3 = 11.5 years (*n* = 27)	No improvements with age
Suppression of interference *(Fruit Stroop Task)*	Hao (2017)	CS	G1 = 7.70 years (*n* = 53) G2 = 9.66 years (*n* = 43) G3 = 11.61 years (*n* = 44)	Significant improvement with age
Suppression of interference *(Stroop-like task)*	Rajan and Bell (2015)	CS	G1 = 6 years (*n* = 35) G2 = 8 years (*n* = 37)	No improvements with age
Suppression of interference *(Attention Network Task)*	Matte-Gagné et al. (2018)	LG	T1 = 1.25 years (*n* = 106) T2 = 2.17 years (*n* = 106) T3 = 6.00 years (*n* = 106) T4 = 7.08 years (*n* = 106) T5 = 7.83 years (*n* = 106) T6 = 8.75 years (*n* = 106)	Significant improvement with age
Suppression of interference *(Attention task)*	Simms et al. (2018)	CS	G1 = 5.5 years (*n* = 25) G2 = 7.5 years (*n* = 29) G3 = 8 years (*n* = 10)	Better performance on this task was positively correlated with age
Suppression of interference *(The Simon task)*	Goriot et al. (2018)	CS	G1 = 4–5 years (*n* = 76) G2 = 8–9 years (*n* = 69) G3 = 11–12 years (*n* = 54)	Better performance on this task was positively correlated with age
Suppression of interference *(Dots spatial conflict task)*	Hoyo et al. (2019)	CS	G1 = 5.82 years (*n* = 43) G2 = 8.96 years (*n* = 43)	Significant improvement with age

*CS, Cross-Sectional; LG, Longitudinal; G, Group; T, Testing Time; RTs, Reaction Time (in milliseconds)*.

a *For a more detailed description of the tasks used to study the different subcomponents of the inhibitory control, see the Supplementary Material*.

##### Suppression of Interference

For this ability we found that 5/9 studies were based on the “Stroop” paradigm (Stroop, 1935), while 2/9 studies used another task that presents two types of trials in which a conflict can occur within the same dimension of the stimulus or stimulus set (e.g., *Attention Network Task)*. Another 3/9 studies reviewed used the *Simon Task* and *Dots Spatial Conflict Task*, where the conflict is produced due to the natural tendency to respond faster when the stimulus and response are ipsilateral—that is, appear on the same side of the screen (Valle-Inclán et al., 1995).

Regarding the 9/19 studies found on the ability to suppress interference, 8/9 studies used a cross-sectional design, while 1/9 used a longitudinal design. This longitudinal study and 6/8 cross-sectional studies found significant improvements in middle childhood (see Table 2). However, 2/8 cross-sectional studies did not observe such significant improvements in this ability with age (Bock et al., 2015; Rajan and Bell, 2015).

#### Cognitive Flexibility

We found 10/44 studies that measured cognitive flexibility, but not all of these 10 studies examined the same subcomponent with the same tasks. A total of 9/10 studies studied set-shifting ability through 2 different types of tasks, while 1/10 measured task-switching through one type of task (for a description see Supplementary Table 2 in Supplementary Material).

##### Set-Shifting

In 6/9 studies the *DCCS* was used and 1/9 study used the *Intra-extra Dimensional Shifting Task* subtest of *CANTAB* battery. In addition, 1/9 study used the *Flanker Task* that was designed primarily to assess the inhibitory control ability, but the authors added a new mixed block with random congruent and incongruent trials to get a switching cost score.

In the studies on set-shifting, 8/9 studies used a cross-sectional design, and 1/9 was a longitudinal study (see Table 3). The results in all 9 studies showed that performance of children improved significantly in middle childhood, especially from age 8 (Bock et al., 2015). Wilson et al. (2018) proposed that children's performance seems to reach an adult level around 12 years.

**Table 3:** Studies on the development of the cognitive flexibility (*n* = 10).

**Subcomponent studied (task)^a^**	**References (year)**	**Design**	**Age groups (*n*)**	**Outcomes**
Set-Shifting *(DCCS)*	Bock et al. (2015)	CS	G1 = 7 years (*n* = 42) G2 = 8.5 years (*n* = 35) G3 = 11.5 years (*n* = 27)	Significant improvement with age, especially from the age of 8
Set-Shifting *(DCCS)*	Chevalier and Blaye (2016)	CS	G1 = 6.4 years (*n* = 25) G2 = 10.5 years (*n* = 28)	Significant improvement with age
Set-Shifting *(DCCS)*	Erb et al. (2017)	CS	G1 = 5–8 years (*n* = 44)	Better performance on this task was positively correlated with age
Set-Shifting *(DCCS)*	Goriot et al. (2018)	CS	G1 = 4–5 years (*n* = 76) G2 = 8–9 years (*n* = 69) G3 = 11–12 years (*n* = 54)	Better performance on this task was positively correlated with age
Set-Shifting *(DCCS)*	Matte-Gagné et al. (2018)	LG	T1 = 1.25 years (*n* = 106) T2= 2.17 years (*n* = 106) T3 = 6.00 years (*n* = 106) T4 = 7.08 years (*n* = 106) T5 = 7.83 years (*n* = 106) T6 = 8.75 years (*n* = 106)	Significant improvement with age
Set-Shifting *(DCCS)*	Perone et al. (2018)	CS	G1 = 3.27 years (*n* = 44) G2 = 4.90 years (*n* = 45) G3 = 5.24 years (*n* = 48) G4 = 9.29 years (*n* = 25)	Better performance on this task was positively correlated with age
Set-Shifting *(DCCS)*	Simms et al. (2018)	CS	G1 = 5.5 years (*n* = 25) G2 = 7.5 years (*n* = 29) G3 = 8 years (*n* = 10)	Better performance on this task was positively correlated with age
Set-Shifting *(Intra-extra dimensional shifting tasks CANTAB)*	Wilson et al. (2018)	CS	G1 = 5 years (*n* = 19) G2 = 6 years (*n* = 15) G3 = 7 years (*n* = 22) G4 = 8 years (*n* = 16) G5 = 9 years (*n* = 22) G6 = 10 years (*n* = 16) G7 = 11 years (*n* = 16)	Significant improvement with age
Set-Shifting *(Flanker Task)*	Ludyga et al. (2018)	CS	G1 = 10–12 years (*n* = 89)	Better performance on this task was positively correlated with age.
Task-Switching *(Dots spatial conflict task)*	Hoyo et al. (2019)	CS	G1 = 5.82 years (*n* = 43) G2 = 8.96 years (*n* = 43)	Significant improvement with age

*CS, Cross-Sectional; LG, Longitudinal; G, Group; T, Testing Time; DCCS, Dimensional Change Card Sort*.

a *For a more detailed description of the tasks used to study the different subcomponents of the cognitive flexibility, see the Supplementary Material*.

##### Task-Switching

We found one study that used a task assessing this component, which was the *Dots Spatial Conflict Task*. The results found in this study which used a cross-sectional design, pointed to a significant improvement in children's performance between 5 and 9 years of age.

#### Summary of Results on the Development of Cool EFs

The present results indicate distinct developmental patterns in the performance of children from 6 to 12 years on cool EF skills. Specifically, we found that the most basic executive components—the ability to inhibit a prepotent response, and set-shifting—both showed continual improvement during this period, reaching a performance similar to that of adults by age 12 (Symeonidou et al., 2016; Wilson et al., 2018). In contrast, the type of inhibitory control that involves a higher cognitive load (suppression of interference caused by distractors) seems to continue to improve beyond 12 years.

Regarding WM, we found that verbal WM and visuospatial WM follow different developmental trajectories. While the development of verbal WM seems to stabilize around 8 years (Matte-Gagné et al., 2018), visuospatial WM continues to develop beyond 12 years (Roberts et al., 2018). The studies reviewed did not find different developmental timelines for static visuospatial WM and dynamic visuospatial WM, but this may be due to the scarcity of investigations on spatial WM.

Results on task-switching and updating abilities must be interpreted with caution as very few studies were found. Of the studies that measured task-switching, we found one study showing significant improvements for updating between 10 and 11 years (Ludyga et al., 2018). However, we cannot conclude that this ability reached a level similar to that of adults by age 11, and more studies are needed to clarify the trajectories of these cool EFs in middle childhood.

### Hot EF Results

#### Decision-Making

Decision-making in situations of uncertainty was studied in 3/44 studies, all of which used a variation of the *Iowa Gambling Task* (IGT–Bechara et al., 1994). In 2/3 studies the original version of the IGT was used, while in 1/3 the authors used a simplified version of IGT (for a description see Supplementary Table 3 in Supplementary Material).

Of the 3 studies on decision-making, 1/3 used a cross-sectional design and the other 2/3 studies used a longitudinal design. The one cross-sectional study reviewed, which used a simplified version of the classic *Iowa Gambling Task*, found significant improvements in children's performance with age, with performance stabilizing after 8 years. In the other 2 longitudinal studies that used more complex versions of the task (similar to those used with adults), it was observed that age-related improvements continued throughout middle childhood and beyond (see Table 4).

**Table 4:** Studies on the development of the decision-making (*n* = 3).

**Component studied (task)^a^**	**References**	**Design**	**Age groups (*n*)**	**Outcomes**
Decision-Making *(Children's Gambling Task)*	Audusseau and Juhel (2015)	CS	G1 = 6.7 years (*n* = 35) G2 = 8.6 years (*n* = 35) G3 =10.7 years (*n* = 35)	Significant improvements with age, although after 8 years they seemed to stabilize
Decision-Making *(Iowa Gambling Task)*	Almy et al. (2018)	LG	T1 = G1:9 years (*n* = 46); G2:13 years (*n* = 73); G3:18 years (*n* = 70) T2 = G1:11 years (*n* = 46); G2:15 years (*n* = 73); G3:20 years (*n* = 70) T3 = G1:13 years (*n* = 46); G2: 17 years (*n* = 73); G3:22 years (*n* = 70) T4 = G1:15 years (*n* = 46); G2: 19 years (*n* = 73); G3:24 years (*n* = 70) T5 = G1:17 years (*n* = 46); G2:21 years (*n* = 73); G3:26 years (*n* = 70)	Significant improvement with age
Decision-Making *(Hungry Donkey Task)*	Lensing and Elsner (2018)	LG	T1 = G1:7.35 years (*n* = 621); G2: 8.90 years (*n* = 975) T2 = G1:8.35 years (*n* = 596); G2: 9.90 years (*n* = 955) T3 = G1:10.35 years (*n* = 565); G2: 11.90 years (*n* = 877)	Significant improvement with age

*CS, Cross-Sectional; LG- Longitudinal; G- Group; T- Testing Time*.

a *For a more detailed description of the tasks used to study the different subcomponents of the Decision-Making, see the Supplementary Material*.

#### Delay of Gratification

We found 3/44 studies that examined delay of gratification. Of these, 2/3 studies used two tasks based on the classic *Marshmallow Task* (Mischel, 1957), and 1/3 study used a *Delay Discounting Task* (Myerson et al., 2001; Scheres et al., 2006) (for a description see Supplementary Table 3 in Supplementary Material).

For delay of gratification, all 3 studies reviewed used a cross-sectional (see Table 5). The results of 2/3 studies that used a task similar to *Marshmallow Task* did not show significant improvement during middle childhood, as both studies found a ceiling effect on performance at 7 years. The 1/3 cross-sectional study that used a *Delay Discounting Task* found significant improvements between 6 and 12 years, followed by a decreased tendency in middle childhood to devalue high-value rewards that involve long wait times.

**Table 5:** Studies on the development of the delay of gratification (*n* = 3).

**Component studied (task)^a^**	**References**	**Design**	**Age groups (n)**	**Outcomes**
Delay of gratification *(Delay of Gratification Task)*	Hao (2017)	CS	G1 = 7.70 years (*n* = 53) G2 = 9.66 years (*n* = 43) G3 = 11.61 years (*n* = 44)	No improvements with age
Delay of gratification *(The gift delay task from the CANTAB)*	Wilson et al. (2018)	CS	G1 = 5 years (*n* = 19) G2 = 6 years (*n* = 15) G3 = 7 years (*n* = 22) G4 = 8 years (*n* = 16) G5 = 9 years (*n* = 22) G6 = 10 years (*n* = 16) G7 = 11 years (*n* = 16)	No improvements with age
Delay of gratification *[An intertemporal choice task (ICT)]*	Steinbeis et al. (2016)	CS	G1 = 6.6–12.7 years (*n* = 20)	Better performance on this task was positively correlated with age

*CS, Cross-Sectional; G, Group*.

a *For a more detailed description of the tasks used to study the different subcomponents of the Delay of Gratification, see the Supplementary Material*.

#### Theory of Mind

As mentioned above, ToM ability was investigated in 16/44 studies found in this review, and as with other EFs, not all ToM subcomponents or abilities were investigated equally, or with the same tasks (for a description see Supplementary Table 3 in Supplementary Material). In this review we found that ToM is a heterogeneous and complex construct that it is usually assessed through 4 different types of tasks, each of which reflect distinct aspects of ToM ability.

The first type of task requires the understanding of false beliefs (FB) of the first and second order. Regarding this aspect of ToM, we found a total of 9/16 studies, which used 5 different tasks to assess the understanding of FBs. In 3/9 studies the tasks included the “location change” paradigm (Wimmer and Perner, 1983), while in another 3/9 studies the tasks were based on the “unexpected content” paradigm (Gopnik and Astington, 1988). In 1/9 study authors used the *Appearance/Reality Task* and in 4/9 studies the tasks involved either the *Interpretive Restricted-View Tasks, Interpretive Ambiguous Figure Task, Interpretive ToM Task, Director Task* or *ToM Task*. In 5/9 studies second-order FBs tasks were used, including the *Second-order FB Task, Ice Cream Truck Story Task, Birthday Puppy Story Task or Affective Second-order FB Task*.

The developmental results found on the 9/9 cross-sectional studies about understanding false beliefs, showed that understanding of FBs improves between 6 and 12 years (see Table 6). However, Chaplin and Norton (2015) noted in their study of children aged 3-12 those improvements are most significant between 5 and 6 years. Results of studies examining second-order FBs showed a later development, with significant improvement between ages 6 and 9, and stabilizing after age 9 (Hayward and Homer, 2017).

**Table 6:** Studies on the development of the ToM (*n* = 16).

**Aspect of ToM studied (task)^a^**	**References**	**Design**	**Age groups (*n*)**	**Outcomes**
Understanding of FBs *(The Sally and Anne FB Task; Unexpected Container Test and; The Duck and Lion Social Test)*	Chaplin and Norton (2015)	CS	G1 = 3 years G2 = 4 years G3 = 5 years G4 = 6 years G5 = 7 years G6 = 8 years G7 = 9 years G8 = 10 years G9 = 11 years G10 = 12 years (total *n* = 159)	Significant improvements with age in all tasks, specially between 5 and 6 years-old
Understanding of FBs *(First-Order FB Task; Second-Order FB Task and; Birthday Puppy Story Task)*	Gómez-Garibello and Talwar (2015)	CS	G1 = 6–9 years (*n* = 426)	Significant improvement with age
Understanding of FBs *(Deceptive Container Tasks; Sally-Anne Task; and Second-Order FB Stories, Affective Second-Order FB Stories)* Understanding non-literal senses: irony, lies and white lies *(Hidden Emotion Task)*	Hoyo et al. (2019)	CS	G1 = 5.82 years (*n* = 43) G2 = 8.96 years (*n* = 43)	Significant improvement with age for both abilities
Understanding of FBs: Study 1: *Unexpected Contents Task; Appearance/Reality Task and; Sandbox Task* Study 2: *Unexpected Contents Task; Appearance/Reality Task; Sandbox Task and; Ice Cream Truck Story Task*	Mahy et al. (2017)	CS	Study 1: G1 = 3.57 years (*n* = 20) G2 = 4.39 years (*n* = 21) G3 = 5.58 years (*n* = 18) G4 = 6.53 years (*n* = 19) G5 = 7.39 years (*n* = 18) Study 2: G1 = 3.51 years (*n* = 18) G2 = 4.35 years (*n* = 18) G3 = 5.56 years (*n* = 18) G4 = 6.56 years (*n* = 18) G5 = 7.51 years (*n* = 18)	In Study 1, better performance on all these tasks was positively correlated with age In Study 2, better performance on all these tasks was positively correlated with age
Understanding of FBs *(Interpretive theory of mind task)*	Kennedy et al. (2015)	CS	G1 = 4.98 years (*n* = 65) G2 = 7.00 years (*n* = 62) G3 = 9.04 years (*n* = 65)	Significant improvement with age
Understanding of FBs *(The director task)*	Symeonidou et al. (2016)	CS	G1 = 11.2 years (*n* = 14) G2 = 16.2 years (*n* = 28) G3 = 23 years (*n* = 23)	Significant improvement with age
Understanding of FBs *(The ToM Task)*	Wang et al. (2016)	CS	G1 = 7.9 years (*n* = 39) G2 = 9.9 years (*n* = 56)	Significant improvement with age
Understanding of FBs *(Second-Order FB Tasks; Interpretive Ambiguous Figure Tasks and; Interpretive Restricted-View Tasks)* Attribution of emotional states in others *(Reading-the-Mind-in-the-Eyes Task)* Faux Pas understanding *(Faux Pas Test)*	Hayward and Homer (2017)	CS	G1 = 7–8 years (*n* = 37) G2 = 9–10 years (*n* = 37) G3 = 11–12 years (*n* = 38)	For the ability of understanding of FBs there were significant improvements with age for all tasks, stabilizing the improvements at 9 years of age in *the second-order FB task* Also, for the attribution of emotional states in others, there were significant improvements with age However, for the ability of understanding Faux Pas there were not significant improvements with age
Understanding of FBs *(Ice Cream Truck Story Task; Birthday Puppy Story Task and;* understanding non-literal senses: irony, lies and white lies *(Strange Stories Task)*	Bock et al. (2015)	CS	G1 = 7 years (*n* = 42) G2 = 8–9 years (*n* = 35) G3 = 10–12 years (*n* = 27)	Significant improvements between 6 and 9 years for the ability of understanding of FBs task. But for the ability of understanding non-literal senses the performance continues to improve until the age of 12
Understanding non-literal senses: irony, lies, and white lies *(Strange Stories Task)*	Lecce et al. (2019)	CS	G1 = 9.6 years (*n* = 62) G2 = 10.5 years (*n* = 48) G3 = 11.5 years (*n* = 51) G4 = 12.4 years (*n* = 56)	Significant improvement with age that begins to stabilize after the age of 10
Understanding non-literal senses: irony, lies, and white lies *(strange stories Task)*	Wilson et al. (2018)	CS	G1 = 5–5.11 years (*n* = 19) G2 = 6–6.11 years (*n* = 15) G3 = 7–7.11 years (*n* = 22) G4 = 8–8.11 years (*n* = 16) G5 = 9–9.11 years (*n* = 22) G6 = 10–10.11 years (*n* = 16) G7 = 11–12.11 years (*n* = 16)	Significant improvement with age that begins to stabilize after the age of 10
Attribution of emotional states in others *(Recognition of basic emotions Task; ToM Storybooks)*	Bulgarelli et al. (2015)	CS	G1 = 3.83 years (*n* = 206) G2 = 5.58 years (*n* = 243) G3 = 7.5 years (*n* = 232)	Significant improvements between 6 and 8 years old only in the *ToM Storybooks*, but not for the Recognition of basic emotion Task due to a possible ceiling effect
Attribution of emotional states in others *(The ToM Task)*	Holl et al. (2018)	LG	T1 = 8.36 years (*n* = 1,657) T2 = 9.12 years (*n* = 1,611) T3 = 11.07 years (*n* = 1,501)	Significant improvement with age
Attribution of emotional states in others *(Past-To-Future Reasoning Task)*	Lagattuta et al. (2016)	CS	G1 = 4–5 years (*n* = 63) G2 = 6–7 years (*n* = 66) G3 = 8–10 years (*n* = 87) G4 = 20 years (*n* = 64)	Group 3 and G4 formed more valence-aligned judgments about how people would think, feel, and act compared with G1 and G2
Attribution of emotional states in others *(Past-To-Future Reasoning Task)*	Lagattuta et al. (2018)	CS	G1 = 4–5 years (*n* = 62) G2 = 6–7 years (*n* = 63) G3 = 8–10 years (*n* = 86) G4 = 20.57 years (*n* = 63)	Significant improvement with age, that begins to stabilize after the age of 8
Attribution of emotional states in others *(Tom Task)*	Brandone and Klimek (2018)	CS	G1 = 8–10 years (*n* = 46) G2 = 11–15 years (*n* = 46) G3 = 18.6 years (*n* = 46) G4 = 34.4 years (*n* = 104)	No improvements with age

*CS, Cross-Sectional; LG, Longitudinal; G, Group; T, Testing Time; ToM, Theory of Mind; FB, False Beliefs*.

a *For a more detailed description of the tasks used to study the different aspects of the ToM, see the Supplementary Material*.

The second type of task that was found to assess ToM was understanding non-literal senses: irony, lies, and white lies. This type of task was found in 4/16 studies. To evaluate this aspect of ToM authors used two different tasks: 3/4 studies used the *Strange Stories Task*, and another 1/4 study used *Hidden Emotion Task*.

All these studies followed a cross-sectional design, and showed that the comprehension of non-literal senses improves with age (see Table 6). However, those using the *Strange Stories Task*, involving metaphorical language such as sarcasm and persuasion, found that this third level of ToM began to stabilize from age 10 (Wilson et al., 2018; Lecce et al., 2019).

The third type of ToM tasks was the attribution of emotional states in others. We found 6/16 that focused on this aspect of ToM using 5 different tasks: 1/6 studies used the *Recognition of basic emotions task;* another 1/6 studies used the *Reading-The-Mind-In-The-Eyes Task*; and another 1/6 studies used the *ToM Storybooks Battery*. The *Past-to-Future Reasoning Task* and *Tom Task* were used in 3/6 studies. Only 1/6 study used the *ToM Task* requiring inference and control of negative emotions.

Results regarding the attribution of emotional states in others, were mixed (see Table 6). In 1/6 longitudinal study and 4/6 cross-sectional studies, results showed significant improvement in the performance on the tasks that evaluated this aspect of ToM in children between the ages of 6 and 12 years. However, there were 2/6 cross-sectional studies that did not find significant improvement. In one of these studies the authors pointed to a ceiling effect (Bulgarelli et al., 2015).

And, the fourth type of task which was found in the present review required the understanding of faux pas. This aspect of ToM was evaluated in 1/16 studies with the *Faux Pas Test*. This only study, which followed a cross-sectional design, found no significant improvements in this ability from age 7 to 12 (see Table 6).

#### Summary of Results Regarding Development of Hot EFs

Hot EFs have received much less attention in the literature than cool EFs. Recent findings on the development of hot EFs seem to depend on the types of tasks used to assess them; therefore, results should be interpreted with caution. The available studies comparing performance between children and adults suggest that the hot abilities of decision-making (Almy et al., 2018; Lensing and Elsner, 2018), and delay of gratification (Steinbeis et al., 2016), improve during middle childhood and beyond age 12. For **ToM** as well, the developmental changes observed depended on the type of task used. Results from false belief tasks showed that the understanding of first-order FBs develops between 5 and 6 years (Chaplin and Norton, 2015), followed by second-order FBs between 6 and 9 years (Hayward and Homer, 2017). From 7 to 12 years the understanding of non-literal sense tasks improves (Bock et al., 2015), although it seems that some aspects of pragmatic language, such as understanding sarcasm and persuasion, are not developed until around 10 years (Wilson et al., 2018; Lecce et al., 2019). The attribution of emotional states in others also improves from 6 to 12 years of age. When simpler tasks were used which required only recognizing emotions through facial expression, a ceiling effect was found between 6 and 8 years (Bulgarelli et al., 2015), while tasks that required understanding how people control negative emotions and their consequences, found no improvements in middle childhood—suggesting that development extends into adolescence (Brandone and Klimek, 2018). Finally, the understanding of faux pas was not observed in middle childhood, suggesting that it does not develop until adolescence as well (Hayward and Homer, 2017). The authors pointed out that these latter tasks may not be suitable for studying ToM in middle childhood, since understanding gaffes implies a relatively demanding level of reasoning.

## Discussion

The purpose of this paper was to systematically review current knowledge regarding the development of EFs during middle childhood. Our first aim was to determine which executive components are the most studied in typically developing children between 6 and 12 years of age. Our review of the literature showed that most studies have focused mainly on three cognitive executive components—working memory, inhibitory control and cognitive flexibility—which are considered by many authors as the central components of cognitive EFs from which more complex ones, such as planning, abstract reasoning or creative thinking, develop (Diamond, 2013).

Despite growing interest in the socio-emotional aspects of executive functioning, we found only a very small number of studies that examined the main two components: decision-making and delay of gratification. While theory of mind appeared in many studies and was determined to be related to development of EFs, none of the authors we reviewed considered it to be an executive function *per se*. We propose, however, that the evidence points to ToM as the executive function that allows us to regulate ourselves within a social context. The ability for affective reversal learning, despite—or perhaps due to—being such a basic process, was not found in any study of typically developing children between 6 and 12 years of age. Most studies that examined affective reversal learning in human development focused on the first few months of life (Overman et al., 1996), or on clinical populations (Wegbreit et al., 2016). Thus, it remains unknown whether or how affective reversal learning as a hot EF continues to develop later—for example, whether the need to learn new or fluctuating associations between a stimulus and a reward or punishment occurs in academic or social situations during childhood.

It seems clear that cool and hot EFs follow distinct developmental trajectories during middle childhood. As we discussed at the beginning of the present work, changes observed at the behavioral level in the development of EFs seem to occur in parallel with maturational brain changes, some in particular occurring in regions of PFC (Best et al., 2009), and others depending on the degree of connectivity between frontal and subcortical limbic areas (Happaney et al., 2004).

For cool EFs, research indicates that the most basic executive components of response inhibition and set-shifting show an increase in development at the behavioral level, as seen in increases in response time and accuracy, which coincide with the refined activation of lateral PFC, bilateral ventral PFC, and right dlPFC (Ezekiel et al., 2013). The refinement of cortical regions translates to faster and more specialized activation of these areas for trials in which these executive skills must be activated. The ability for interference suppression, as measured with the Stroop paradigm in neuroimaging studies, shows an improvement between 6 and 12 years that is associated with greater activation of left lateral PFC (Adleman et al., 2002).

The developmental of verbal WM precedes that of visuospatial WM by several years at the behavioral level. The neuroanatomical substrates for WM show increased reliance on the activation of the anterior insular cortex for verbal WM (Rossi et al., 2013), and on prefrontal and parietal regions for visuospatial WM (Klingberg et al., 2002) in children between 6 and 12 years.

Data regarding development for task-switching are scarce at the behavioral level, and lacking in neuroimaging studies as far as we know to date in children between 6 and 12 years of age, making it of great importance to continue researching how this executive component develops during middle childhood. For updating, the few studies found using the n-back paradigm suggest that this ability improves significantly between 10 and 11 years (Ludyga et al., 2018), possibly in association with decreased activation of the prefrontal regions during middle childhood (see meta-analysis Yaple and Arsalidou, 2018).

The development of hot EFs is much less clear. The studies reviewed here suggest that decision-making shows a behavioral improvement during middle childhood that continues beyond 12 years (Almy et al., 2018; Lensing and Elsner, 2018), which is consistent with evidence of a later functional maturation of the vmPFC (Crone and van der Molen, 2007). Similarly, the development of delay of gratification showed improvements at the behavioral level associated with the maturation of right dlPFC, which together with the vmPFC, is involved in choosing greater delayed rewards vs. smaller but immediate rewards (Steinbeis et al., 2016).

With respect to ToM, we found a wide variety of tasks in this review, as it is quite a broad and heterogeneous construct (Hayward and Homer, 2017). However, because a milestone that is purported to mark the appearance of ToM is the understanding of first-order FB, most of the studies reviewed have focused on this ability (Wimmer and Perner, 1983). While numerous studies have shown that children demonstrate this ability from the age of 4 years, the studies reviewed here showed that it may continue to improve beyond 6 years (Chaplin and Norton, 2015). Neuroimaging studies have demonstrated that brain regions associated with understanding first-order FBs, such as mPFC, show a decrease in activation during middle childhood (Mukerji et al., 2019). One possible interpretation of these collective findings might be that a reduction in activation of MPFC corresponds to an increase in efficiency as first-order FB understanding is strengthened, or perhaps a shift in strategy requiring different activation patterns, for those aspects of behavior that do continue to develop beyond age 6. Future investigations may require tasks that differentiate between processes related to ToM before age 6, and after, as well as changes in the underlying neural substrates.

When considered together, the available evidence on cognitive and socio-emotional components of EFs during middle childhood suggests that while their developmental trajectories differ, they are not totally independent. In fact, the few studies reviewed here that have taken into consideration the socio-emotional aspects of EFs, have observed a possible close relationship between hot and cool components. Specifically, some studies have found significant correlations between WM and decision-making (Lensing and Elsner, 2018). Others (Hao, 2017) have found that delay of gratification is an affective (hot) type of inhibitory control, that differs from its cool counterpoint—mainly in its motivational and emotional aspects, and its involvement in highly rewarding situations (Simpson and Carroll, 2019).

ToM is an interesting set of abilities that seems to have a potentially important role in illuminating the relationship both between cool and hot EFs, and between individual vs. social skills in childhood. While ToM falls especially under the umbrella of hot EFs (e.g., Zimmerman et al., 2016), some studies found that ToM tasks could be used to show significant correlations between WM and cognitive flexibility (Hoyo et al., 2019). As with other hot EFs, ToM is involved in situations that generate emotion and motivation, as well as tension between immediate gratification and greater long-term reward. However, ToM seems to be a key to understanding these aspects of behavior in social contexts, which involve regulating oneself in relation to others.

Although the studies examined in this review have shown that cognitive and socio-emotional components of EFs follow particular developmental trajectories during childhood, they also suggest that the developmental trajectories observed are sensitive to the type of tasks used to assess each component. It is important to note that, although there are studies comparing performance on these tasks between different age groups, the studies reviewed offered only correlational analyses between cool and hot EFs. Understanding the nature of the relationship between cool and hot EFs will require more controlled studies of both cross-sectional and longitudinal design, in which the same tasks are used for all age groups.

A few final limitations are notable from the present review. First, a reason for caution regarding the results for hot EFs, as mentioned earlier, is the small number of studies that we found evaluating these components. More studies are still needed to jointly assess cognitive and socio-emotional components of EFs in middle childhood. We also consider it important to mention that while the studies included in this review cover a wide variety of cultures and countries, they do not address possible cultural biases associated with the results. A final limitation of this review is that despite rigorously following the Cochrane guidelines (Higgins and Sally, 2008) and the PRISMA statement (Moher, 2009), the authors cannot guarantee that all relevant data were retrieved. This is because the search did not include other material such as gray literature. Furthermore, it is possible that a selection bias occurred due to language limitations. In this systematic review, only studies published in English or Spanish were selected; although language bias has been shown to have little impact on the results when studies in English are not excluded (Pereira et al., 2018).

## Conclusion

This review summarizes the development of EFs as studied in the literature, and examines them separately as cool and hot components, that follow distinct but likely interdependent developmental trajectories, and reach maturity at different stages of development. The developmental trajectories observed seem to occur in parallel with patterns of maturation in specific areas of the PFC, which also show differences in the rate at which they mature. It is important to emphasize that, although we make this distinction at the conceptual level between cognitive and socio-emotional EF, these components are part of an integrated system where they usually work together (Zelazo and Carlson, 2012). Understanding the distinction between cool and hot EFs, and their integration in development, has important implications for education, clinical practice, and research in the field of executive functions in typical and atypical populations. This executive functioning model has the potential to help inform the distinct roles of cognitive and socio-emotional components of EFs in development in the general population, as well as in a wide variety of neurodevelopmental disorders where deficits in EFs have been found (Tsermentseli and Poland, 2016).

Although it is known that childhood is a period of development where peer relationships and social regulation become increasingly important, we know little about the developmental trajectory of cognitive and socio-emotional components of EFs (especially the latter). Therefore, understanding EFs as presented in this review could help us to understand how children learn to inhibit their own perspective and shift their mental state to take on another's, and then maintain it in order to form an appropriate response. With this systematic review, we hope to provide more knowledge about how cognitive and socio-emotional EFs develop, and an updated synthesis of the tools most widely used to assess executive functioning during middle childhood. This review might also provide a helpful reference in the design of more specific evaluations and interventions focused on this specific age range. This work is necessary for scientists, clinicians, and educators to understand typical development and to help prevent and detect potential developmental problems during childhood.

## Statement of Public Importance

This systematic review offers an integrative theoretical framework of the most studied executive function components. This framework takes into account the importance of socio-emotional executive functions, which children need in order to regulate their behavior and emotions, and respond appropriately to the demands of the environment.

Furthermore, this review provides a synthesis of the most widely used tools to evaluate *cool* and *hot* executive functions, and which of these tools are most sensitive to detecting changes at the behavioral level that occur during middle childhood in executive functions.

## Data Availability Statement

The original contributions generated for the study are included in the article/Supplementary Material, further inquiries can be directed to the corresponding author/s.

## Author Contributions

LF, MD, and JP-S contributed to the conception and design of the study. LF was primarily responsible for the literature search, the study selection process, data extraction, and wrote the first draft of the manuscript. AM helped with the study selection process and data extraction. MD and JP-S supervised the study. All authors contributed to manuscript revision and read and approved the submitted version.

## Conflict of Interest

The authors declare that the research was conducted in the absence of any commercial or financial relationships that could be construed as a potential conflict of interest.

## Publisher's Note

All claims expressed in this article are solely those of the authors and do not necessarily represent those of their affiliated organizations, or those of the publisher, the editors and the reviewers. Any product that may be evaluated in this article, or claim that may be made by its manufacturer, is not guaranteed or endorsed by the publisher.
