# Supplementary material for: Neuropsychological Development of Cool and Hot Executive Functions Between 6 and 12 Years of Age: A Systematic Review
Source: Front Psychol. 2021 Aug 11;12:687337. doi: 10.3389/fpsyg.2021.687337 (PMC8385409; doi:10.3389/fpsyg.2021.687337)
Supplement: Supplementary file 1 [file Table_1.docx]

**SUPPLEMENTARY MATERIAL**

Table 1. NEWCASTLE - OTTAWA QUALITY ASSESSMENT SCALE (adapted for cross-sectional studies).

| **Selection** | | | |  | **Comparability** | **Outcome** | | |  | |
| --- | --- | --- | --- | --- | --- | --- | --- | --- | --- | --- |
| **Authors** | **Representativeness of the sample** | **Sample size** | **Non-respondents** | **Ascertainment of the exposure** | **Based on design and analysis** | | **Assessment of outcome** | **Statistical tests** | **Total (max. 10)** |  |
| Kennedy, 2015 | * | * | * | * | ** | | ** | * | 9 |  |
| Bulgarelli, 2015 | * | * | * | ** | ** | | - | * | 8 |  |
| Holl, 2018 | * | * | * | ** | ** | | - | * | 8 |  |
| Lensing, 2018 | * | * | * | ** | ** | | - | * | 8 |  |
| Lee Swanson, 2015 | * | * | * | ** | ** | | - | * | 8 |  |
| Lagattuta, 2016 | * | * | * | ** | ** | | - | * | 8 |  |
| Matte-Gagné, 2018 | * | * | * | ** | ** | | - | * | 8 |  |
| Mous, 2017 | * | * | * | ** | ** | | - | * | 8 |  |
| Almy, 2018 | * | * | * | * | ** | | - | * | 7 |  |
| Bellaj, 2016 | * | - | * | ** | ** | | - | * | 7 |  |
| Gómez-Garibello, 2015 | * | * | - | ** | ** | | - | * | 7 |  |
| Hao, 2017 | * | * | * | * | ** | | - | * | 7 |  |
| Hayward, 2017 | * | * | * | ** | * | | - | * | 7 |  |
| Lagattuta, 2018 | * | * | * | * | ** | | - | * | 7 |  |
| Lecce, 2019 | * | * | - | ** | ** | | - | * | 7 |  |
| Ludyga, 2018 | - | * | * | ** | ** | | - | * | 7 |  |
| Roberts, 2015 | * | * | * | ** | ** | | - | - | 7 |  |
| Simms, 2018 | * | * | * | ** | * | | - | * | 7 |  |
| Audusseau, 2015 | - | * | - | * | * | | ** | * | 6 |  |
| Barriga-Paulino, 2017 | - | * | * | * | ** | | - | * | 6 |  |
| Bock, 2015 | * | * | * | * | ** | | - | - | 6 |  |
| Brandone, 2018 | * | * | - | * | ** | | - | * | 6 |  |
| Chaplin, 2015 | * | * | - | * | ** | | - | * | 6 |  |
| Hoyo, 2019 | * | * | - | * | ** | | - | * | 6 |  |
| Mahy, 2017 | * | * | * | * | * | | - | * | 6 |  |
| Nys, 2018 | - | - | * | ** | ** | | - | * | 6 |  |
| Perone, 2018 | * | * | * | * | * | | - | * | 6 |  |
| Roberts, 2018 | - | * | * | ** | * | | - | * | 6 |  |
| Vogan, 2016 | * | - | * | * | ** | | - | * | 6 |  |
| Aïte. 2016 | - | * | * | * | * | | - | * | 5 |  |
| Arbel, 2018 | * | * | * | * | * | | - | - | 5 |  |
| Goriot, 2018 | * | - | * | * | * | | - | * | 5 |  |
| Kharitonova, 2015 | - | - | * | * | ** | | - | * | 5 |  |
| Rajan, 2015 | - | * | * | ** | * | | - | - | 5 |  |
| Steinbeis, 2016 | - | - | * | * | ** | | - | * | 5 |  |
| Symeonidou, 2016 | - | - | * | * | ** | | - | * | 5 |  |
| Wang, 2016 | - | * | * | * | * | | - | * | 5 |  |
| Wilson, 2018 | - | - | - | ** | ** | | - | * | 5 |  |
| Yang, 2018 | - | * | * | * | * | | - | * | 5 |  |
| Chevalier, 2016 | * | - | - | * | * | | - | * | 4 |  |
| Erb, 2017 | - | * | - | * | * | | - | * | 4 |  |
| Plebanek, 2019 | - | - | * | * | * | | - | * | 4 |  |
| Barry, 2018 | - | * | - | - | * | | - | * | 3 |  |
| Pailian, 2016 | - | - | * | * | * | | - | * | 3 |  |

**Note**: The stars (*) represent scores. A study can be awarded a maximum of one star for the following categories: representativeness of sample, sample size, non-respondents, statistical tests. A maximum of two stars can be awarded for the following categories: ascertainment of the exposure, based on design and analysis, assessment outcome. Higher scores reflect greater methodological quality with 10 being the highest score that can be achieved.

**Table 2.** The main tasks used to assess cool EFs between 6 and 12 years**.**

| **Working Memory** | | | |
| --- | --- | --- | --- |
| **Subcomponent** | **Task** | **Study** | **Description** |
| Verbal WM | DMTS tasks *(maintain and manipulate*): The Letter Matching task. | Vogan et al. (2016) | Delayed Match-To-Sample tasks (DMTS; Daniel et al., 2016) consist of three phases: sample, delay, and choice. First, the participants are presented with a stimulus (the sample) that they must memorize. Then, a delay is introduced (lasting from seconds to several minutes) in which no stimulus is present. During the delay phase, the participants must maintain a representation of the sample. Finally, in the choice phase, stimuli are again presented on the screen, and the participant must indicate whether the stimuli are the same as the previous ones presented. In the *Letter Matching task* participants must decide whether the global capital letter “A” contains some of the letters considered relevant (A, B, E, H). After a delay time, where the stimulus set disappears, they indicate if the new a global capital letter “A” contains the same relevant letter that they identified previously. |
|  | WM span tasks *(only maintaining*): The Memory for Sentences task. | Kennedy et al. (2015); Lagattuta et al. (2016; 2018) | In WM span tasks, the amplitude of the WM is tested by requiring the participants to see or hear a sequence of stimuli (digits, numbers, letters, words or, phrases) and recall the sequence correctly. Increasingly longer sequences are tested in each trial, saturating the verbal WM and implicating greater participation of the central executive. These tasks measure verbal WM when the set of stimuli can be maintained in the WM by means of subvocal rehearsal. In the *Memory for Sentences task* participants must repeat a sequence of phrases in the same order that they had heard before. |
|  | WM span tasks *(maintain and manipulate*): Digit Span subtest; Forward Digit Span and Backward Digit Recall subtest; Backward digit span task and; Digits Forward Interference task. | Bellaj et al. (2016); Bock et al. (2015); Goriot et al. (2018); Hoyo et al. (2019); Lecce et al. (2019); Lee Swanson et al. (2015); Lensing et al. (2018); Matte-Gagné et al. (2018); Nys et al. (2018); Rajan et al. (2015); Roberts et al. (2015; 2018); Simms et al. (2018) | These WM span tasks can be categorized into two main types: *Forward Digit Span* task, where participants must reproduce a sequence of digits in the same order in which it was presented (e.g., 1, 2, 3, 4, …), and *Backward Digit Span* task, where they must repeat the sequence in the reverse order (e.g., …4, 3, 2, 1). In the *Digit Forward Interference* task, unlike the others, before participants reproduce sequence of stimuli presented in the same order, they must answer a question that is irrelevant to the task. |
| Static visuospatial WM | DMTS task *(only maintaining*): Visual Delayed Match-to-Sample task; Working Memory task; Flicker Change Detection task; Working Memory Capacity task; Spatial Working Memory task and; Change detection task. | Barriga-Paulino et al. (2017); Kharitonova et al. (2015); Pailian et al. (2016); Plebanek et al. (2019); Roberts et al. (2018); Wilson et al. (2018); Yang et al. (2018) | In these tasks a stimulus is presented, or a set of stimuli presented simultaneously. After a delay, the stimulus (set) is presented again on the screen and participants must detect whether there is any change with respect to the location. |
|  | DMTS task *(with interference*): Visual Matrix, and Mapping and Directions tasks and; Dots and Dots Interference subtests from the Working Memory Battery (WOMBAT). | Lee Swanson et al. (2015); Roberts et al. (2018) | These tasks are similar to the one presented above, but now there is a higher cognitive demand by introducing an irrelevant question to the task during the delay time. |
|  | WM span tasks *(maintain and manipulate):* Dots Up subtest from the Working Memory Battery (WOMBAT). | Roberts et al. (2018) | As in verbal WM span tasks, the amplitude of the WM is tested by requiring the participants to see or hear a sequence of stimuli, but in this case, they must recall the spatial locations. These tasks measure visuospatial WM since the set of stimuli cannot be maintained in the WM by means of subvocal rehearsal, but require other strategies to maintain the information in the WM. In the *Dots Up subtest* a set of points located within a matrix were presented simultaneously. Then participants were shown that same matrix but with empty grids, and they had to indicate location by “clicking” on the grid located one position up from the grid where each point was presented. |
| Dynamic visuospatial WM | WM span tasks *(only maintaining*): Dots Sequence and Dots Sequence Interference subtest from the Working Memory Battery (WOMBAT). | Roberts et al. (2018) | In the *Dots Sequence subtest*, a series of points appear sequentially within a matrix, and participants are asked to indicate the order in which the dots appeared. In the *Dots Sequence Interference subtest*, during the delay participants are presented with an irrelevant question. |
|  | WM span tasks *(maintain and manipulate*): The Corsi test; Location memory task; Spatial Span Test and; Dots Sequence Backward subtest*.* | Bellaj et al. (2016); Bock et al. (2015); Nys et al. (2018); Roberts et al. (2018) | In these tasks participants must point to the grid where stimuli previously appeared, indicating the reverse order in which they were presented. |
| Updating | “N-back” paradigm | Lee Swanson et al. (2015); Ludyga et al. (2018) | In tasks based on this paradigm a sequence of items (colored shapes or numbers) is presented in succession (for example: A, F, G…). On each trial, participants must answer whether the last stimulus presented is identical to the one that appeared 2 trials before. |
| **Inhibitory Control** | | | |
| **Subcomponent** | **Task** | **Study** | **Description** |
| Inhibit a predominant response | Go / No Go Task and; Stop-Signal Task | Arbel et al. (2018); Barry et al. (2018); Steinbeis et al. (2016); Symeonidou et al. (2016); Wilson et al. (2018) | In this type of tasks, a particular response becomes automated when most trials require this response to the presence of a certain cue (“Go” trials). In some trials, participants are required to inhibit such an automated response when another cue appears (“No Go” or “Stop Sign” trials). The latter type of trial implies a greater executive demand since it requires suppressing a predominant motor response. |
|  | Happy-Sad Task and Day-Night Task; Grass/ Snow Task and; Head - Shoulders - Knees - Toes Task and; Response Set Task | Kennedy et al. (2015); Lagattuta et al. (2016, 2018); Mahy et al. (2017); Mous et al. (2017) | In these tasks participants must inhibit the predominant response of the name of some stimulus, and instead say the opposite (for example, saying “happy” in front of a picture of sad face; or touching one’s shoulders when the experimenter said to touch knees, and the contrary). |
| Suppression of the interference | “Stroop” paradigm | Aïte et al. (2016); Bellaj et al. (2016); Bock et al. (2015); Hao (2017); Rajan et al. (2015) | In the tasks based on this paradigm a bivalent stimulus is presented that can be labelled according to two conflicting dimensions (for example, the word “GREEN” written in blue ink, or the picture of a banana colored blue). Participants are asked to respond by focusing on one dimension of the stimulus (i.e., say the color of the ink, or say the typical color of the banana), while ignoring the other irrelevant dimension (the meaning of the word, or the color in which the fruit is shown). Therefore, they must suppress the interference caused by the irrelevant dimension (the word “GREEN” or; the color shown). |
|  | Attention Network Task | Matte-Gagné et al. (2018); Simms et al. (2018) | In this task, five identical fish are presented, and participants are asked to indicate the direction in which the central fish is pointing. The other fish can point in the same direction as the central fish (congruent trial), or in the opposite direction (incongruent trial). In incongruent trials the participants must focus their attention on the central stimulus and suppress the interference caused by other distracting stimuli. |
|  | Simon Task and; Dots Spatial Conflict Task | Aïte et al. (2016); Goriot et al. (2018); Hoyo et al. (2019) | In these tasks a stimulus is presented on the left or right of the screen. Participants must press the left button on the keyboard when the stimulus is of a certain color and/or shape (for example, a red circle). When the stimulus is, for example, a blue circle, they must press the button located on the right side of the keyboard. In congruent trials the side on which the stimulus appears on the screen and the side where the response key is located are the same. However, in incongruent trials there is a conflict between the relevant dimension for the task (responding according to the color of the stimulus) and the irrelevant dimension (the spatial location of the stimulus with respect to the fixation point), requiring the ability to suppress the interference of distractors. |
| **Cognitive Flexibility** | | | |
| **Subcomponent** | **Task** | **Study** | **Description** |
| Set-shifting | DCCS | Bock et al. (2015); Chevalier et al. (2016); Erb et al. (2017); Goriot et al. (2018); Matte-Gagné et al. (2018); Perone et al. (2018); Simms et al. (2018) | In this task participants are presented with a target stimulus that can vary in color (red or blue) and in shape (picture of a rabbit or boat), and two reference stimuli that each share one characteristic with the target (color or shape). Depending on the instruction on each trial (“pay attention to color” or “pay attention to shape”), the participant must focus attention on the indicated characteristic and choose the category where the target stimulus (for example, a red rabbit) matches that characteristic. |
|  | Intra-extra Dimensional Shifting Task subtest of CANTAB battery | Wilson et al. (2018) | The main difference between this task and the *DCCS* is that the *Intra-extra Dimensional Shifting* task provides feedback after each trial, while *DCCS* does not. |
|  | Flanker Task | Ludyga et al. (2018) | Participants first perform a block where congruent trials (where there is no interference between the target stimulus and the distractors), and incongruent trials (where the distractors are different from the target stimulus), are randomly combined. The second part of the task consists of a block of trials in which the color of the stimuli indicate the type of response that the participants must give. For example, when the stimuli are blue, participants must ignore the direction indicated by the central stimulus and respond to the flanking stimuli. However, when the stimuli are black, they must follow the originally trained instruction and respond to the stimulus at the center. As a measure of cognitive flexibility, the overall switching costs are calculated by subtracting the mean RTs in the standard block trials from the mean RTs in the mixed block trials. |
| Task-switching | Dots Spatial Conflict Task | Hoyo et al. (2019) | In this task, the authors obtain a measure of task-switching by using a block of only congruent trials (when the side of the screen on which the stimulus appears and the side where the response key is located match), and another block of only incongruent trials (when the location of the stimulus on the screen and the location of the response key are different). A measure of task-switching ability is obtained by comparing performance on the mixed block (which included both congruent and incongruent trials) with the simple blocks (only congruent or incongruent). |

**Note**: WM- Working Memory; DMTS- Delayed Match-To-Sample tasks; DCCS- Dimensional Change Card Sort.

**Table 3.** The main tasks used to assess hot EFs between 6 and 12 years**.**

| **Decision-Making** **in situations of uncertainty** | | | |
| --- | --- | --- | --- |
| **Subcomponents** | **Task** | **Study** | **Description** |
|  | Children’s Gambling Task | Audusseau et al. (2015) | The main difference from the other two decision-making tasks is that in the classic *Iowa Gambling Task* and the *Hungry Donkey Task* participants have to choose between 4 options, while the *Children’s Gambling Task* is a simplified version that includes only two options to choose from (one advantageous and one disadvantageous). |
|  | Iowa Gambling Task; Hungry Donkey Task | Almy et al. (2018); Lensing et al. (2018) | In this paradigm, participants must choose between four apparently equal options. However two of them (A and B) are disadvantageous because their choice is followed by greater losses than the other two options considered advantageous (C and D). During the task, the participants are unaware of this information, and are only asked to achieve the highest possible score. This paradigm records the “Net Score” as a measure of decision-making capacity, which reflects the difference between the number of advantageous choices compared to disadvantageous choices. Thus, a better ability to make decisions is reflected by a greater number of advantageous choices at the end of the task. |
| **Delay of Gratification** | | | |
| **Subcomponents** | **Task** | **Study** | **Description** |
|  | Marshmallow tasks: *Delay of Gratification Task; The gift delay task from the CANTAB* | Hao et al. (2017); Wilson et al. (2018) | Variants of the Marshmallow task (e.g., *Gift Delay task* and *Delay of Gratification task*) record the length of time participants are willing to wait to get a large reward (for example, two candies), rather than getting a minor reward (one candy) that requires no waiting time. |
|  | Delay Discounting tasks: *An intertemporal choice task (ICT)* | Steinbeis et al. (2016) | In the *Intertemporal Choice Task* based on Delay Discounting tasks, on each trial participants must choose between two options. The first is an immediate reward that does not require waiting, but that is of a lower value, and can vary randomly throughout the trials (2, 4 or 6 coins). The second is a higher reward that always has the same value (8 coins), but that is associated with a waiting time that can vary through the trials of the task (4, 6, 28 or 56 days). The ability to delay gratification is reflected in the “area under the discount curve” (AUC). This measure shows the tendency of the participants to discount the value that the delayed reward has for each participant (and which is therefore subjective), as the time they have to wait to get that reward increases. Thus, the subjective value of the reward is measured by the value of the immediate reward (for example, 4 coins now) against which the participant stopped preferring the reward of greater value (8 coins after 56 days), because it was determined not to be worth waiting for. |
| **Theory of Mind** | | | |
| **Subcomponents** | **Task** | **Study** | **Description** |
| Understanding of false beliefs (FB) of the first and second order | Paradigm of “location-change” | Chaplin et al. (2015); Gómez-Garibello et al. (2015); Hoyo et al. (2019) | Participants must put themselves in the place of a character A who has not seen how another character B has changed the location of a target or has stopped doing a specific action, and therefore whose belief does not correspond to reality. |
|  | Paradigm of “unexpected content” | Chaplin et al. (2015); Hoyo et al. (2019); Mahy et al. (2017) | Participants may have an erroneous belief regarding the content of a box (for example, cookies) when the content does not correspond to their expectations based on in previous experiences (can contain pencils). |
|  | Appearance / Reality Task | Mahy et al. (2017) | This is similar to the procedure followed in the “unexpected content” paradigm, except participants must indicate what deceptive stimulus was visually presented to them (for example, a sponge that looks like a rock). |
|  | Interpretive Restricted-View Tasks; Interpretive Ambiguous Figure Task; Interpretive ToM Task; Director Task and; ToM Task. | Hayward et al. (2017); Kennedy et al. (2015); Symeonidou et al. (2016); Wang et al. (2016) | This task involves make inferences about what the character will think according to the limited view with respect to the stimulus presented. |
|  | Second-order FB Task; Ice Cream Truck Story Task and; Birthday Puppy Story Task; Affective Second-order FB Task | Bock et al. (2015); Gómez-Garibello et al. (2015); Hayward et al. (2017); Hoyo et al. (2019); Mahy et al. (2017) | Second-order TB tasks imply the understanding that a character A may have erroneous knowledge about the belief of another character B. For example, Maria does not know that Pablo has just found out that the ice cream man has gone to church, so she believes that Pablo will look for the ice cream man where they saw him in the morning (in the park). The main difference between affective second-order FB tasks and the other is that in this type of task participants have to infer a character A’s belief about the emotion of another character B, instead of a belief. |
| Understanding non-literal senses: irony, lies and white lies | Strange Stories Task | Bock et al. (2015); Lecce et al. (2019); Wilson et al. (2018) | Participants must explain why the protagonist said something incorrect. Scores are awarded based on the degree to which children respond in terms of the character’s mental states (beliefs, emotions, desires), rather than their physical or situational attributes. |
|  | Hidden Emotion Task | Hoyo et al. (2019) | Participants must infer the emotional mental state of a character within a context (for example, sadness for not having received the desired gift), and to explain why despite feeling this emotion the character shows a different emotion (smiles at grandmother for the gift). Therefore, this task implies the understanding of a white lie. |
| Attribution of emotional states in others | Recognition of basic emotions Task | Bulgarelli et al. (2015) | There are five emotion recognition tasks: happy, scared, angry, sad and surprised. The child is presented with five situational descriptions. It has to choose the appropriate face and provide the correct emotion label. To avoid a response bias, the presentation order of the faces varied. Example task (see Fig. 1): ‘Sam has won shooting marbles. He has won the most beautiful marble.’ Questions: (1) Choose the face that matches. (emotion recognition), (2) How does he look? (emotion naming), (3) How come Sam is feeling happy? |
|  | Reading-The-Mind-In-The-Eyes Task and | Hayward et al. (2017) | Participants are presented with a series of images of eyes, providing as little information about the individual or social context as possible. They must infer the emotional or mental state of the person (joy, sadness, surprise) and select the appropriate affective term that matches the image of eyes. |
|  | ToM Storybooks Battery | Bulgarelli et al. (2015) | Participants must distinguish between physical and mental entities, understand that seeing something implies having knowledge about it, and understand that desires and beliefs can affect behavior. |
|  | Past-to-Future Reasoning Task and; Tom Task | Holl et al. (2018); Lagattuta et al. (2016, 2018) | In both tasks participants must infer the mental state of a character within a specific context. |
|  | ToM Task | Brandone et al. (2018) | Children must infer negative emotions that other people experience (for example, Maria was angry after losing a basketball game) and their ability to control them in a conflict situation with another person (responding aggressively or being calm). |
| Faux pas understanding | Faux Pas Test | Hayward et al. (2017) | This task requires understanding the faux pas that occur when a character says something without considering that the listener might not want to hear or know, and that usually has negative consequences that the speaker never intended (for example, Maria says “*I had not met this boy yet*”, to a mother, whose child is actually a girl). In this task, participants must detect the difference between the knowledge of the speaker and the listener, and assess the emotional impact of the faux pas on the listener. |

**Note**: ToM- Theory of Mind; FB- False Beliefs.
